# The bHLH Repressor Deadpan Regulates the Self-renewal and Specification of *Drosophila* Larval Neural Stem Cells Independently of Notch

**DOI:** 10.1371/journal.pone.0046724

**Published:** 2012-10-08

**Authors:** Sijun Zhu, Jill Wildonger, Suzanne Barshow, Susan Younger, Yaling Huang, Tzumin Lee

**Affiliations:** 1 Department of Physiology, University of California San Francisco, San Francisco, California, United States of America; 2 Howard Hughes Medical Institute, University of California San Francisco, San Francisco, California, United States of America; 3 Janelia Farm Research Campus, Howard Hughes Medical Institute, Ashburn, Virginia, United States of America; University of Nebraska Medical Center, United States of America

## Abstract

Neural stem cells (NSCs) are able to self-renew while giving rise to neurons and glia that comprise a functional nervous system. However, how NSC self-renewal is maintained is not well understood. Using the *Drosophila* larval NSCs called neuroblasts (NBs) as a model, we demonstrate that the Hairy and Enhancer-of-Split (Hes) family protein Deadpan (Dpn) plays important roles in NB self-renewal and specification. The loss of Dpn leads to the premature loss of NBs and truncated NB lineages, a process likely mediated by the homeobox protein Prospero (Pros). Conversely, ectopic/over-expression of Dpn promotes ectopic self-renewing divisions and maintains NB self-renewal into adulthood. In type II NBs, which generate transit amplifying intermediate neural progenitors (INPs) like mammalian NSCs, the loss of Dpn results in ectopic expression of type I NB markers Asense (Ase) and Pros before these type II NBs are lost at early larval stages. Our results also show that knockdown of Notch leads to ectopic Ase expression in type II NBs and the premature loss of type II NBs. Significantly, *dpn* expression is unchanged in these transformed NBs. Furthermore, the loss of Dpn does not inhibit the over-proliferation of type II NBs and immature INPs caused by over-expression of activated Notch. Our data suggest that Dpn plays important roles in maintaining NB self-renewal and specification of type II NBs in larval brains and that Dpn and Notch function independently in regulating type II NB proliferation and specification.

## Introduction

Neural stem cells (NSCs), like other stem cells, maintain their undifferentiated proliferative status while undergoing many rounds of cell division to produce a diverse array of neurons and glia. Proliferating NSCs are maintained through symmetric divisions, which expand the NSC pool, as well as self-renewing asymmetric cell divisions, which produce one daughter that becomes a NSC and another daughter with limited proliferative potential that will produce differentiated progeny. Maintaining the self-renewal of NSCs is critical for the proper formation and homeostasis of the nervous system. Premature termination of NSC self-renewal can lead to the reduction or loss of particular cell types, whereas continued or increased NSC self-renewal can lead to tumor formation. Hence, deciphering the mechanisms underlying the balance between NSC self-renewal and neuronal differentiation is important for understanding normal neurogenesis as well as the pathology of diseases that result from perturbations in NSC self-renewal. However, the mechanisms maintaining NSC self-renewal and how NSC proliferation and differentiation are balanced are just beginning to be understood.

**Figure 1:**
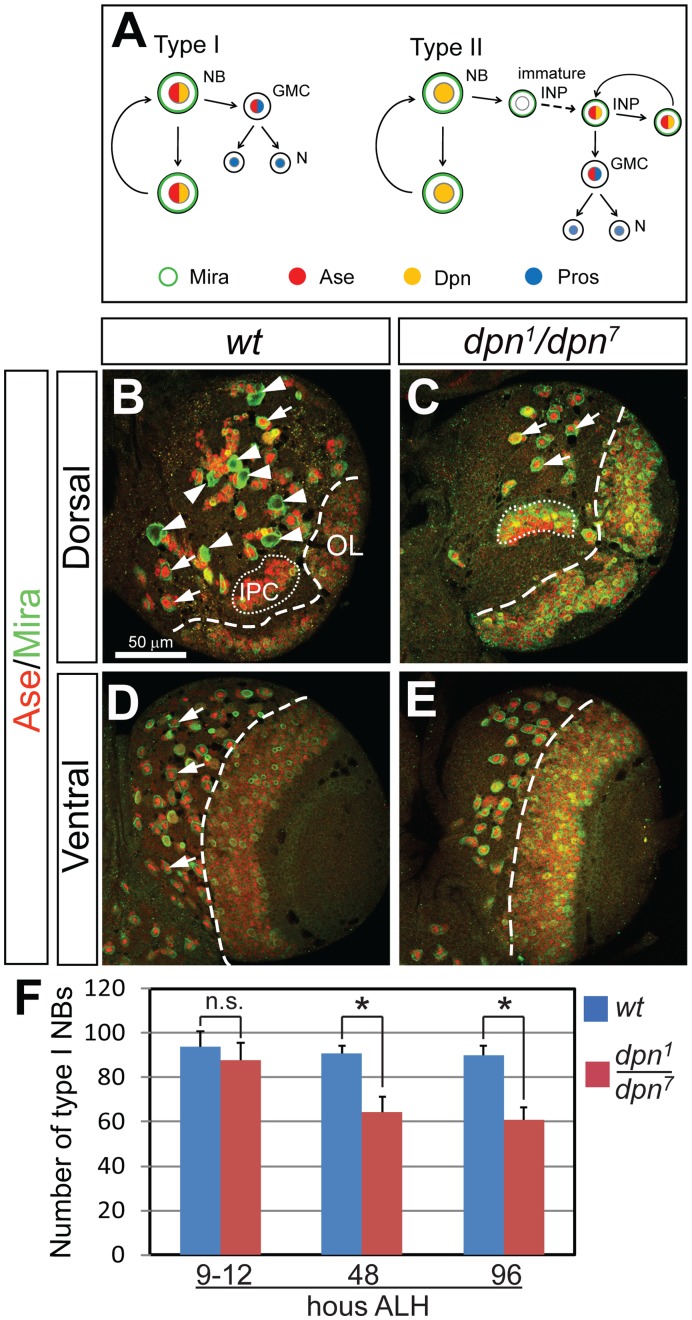
Loss of Dpn leads to a complete loss of type II NBs and a dramatic reduction of type I NBs at the late 3^rd^ instar larval stage. (A) Schematic diagram of the division patterns of type I (left) and type II (right) neuroblast lineages. NB, neuroblast; GMC, ganglion mother cell; N, neuron; INP, intermediate neural progenitor. Dpn is expressed in both type I and type II NBs as well as mature INPs in the type II lineages. Ase is expressed in type I NBs, GMCs and mature INPs, but not in the type II NBs. Drawing is based on Boone and Doe, 2008. (B–C) Dorsal views of larval brains showing that *ase* ^-^ type II NBs (arrowheads in (C)) are completely missing in *dpn^1^/dpn^7^* mutants and only type I NBs (e.g. arrows in (C)) remain on the dorsal side of the brain at 4 days after larval hatching (ALH). (D–E) Ventral side of the larval brains showing that type I NBs (arrows) are reduced in *dpn* loss-of-function mutants at 4 days ALH. (F) Quantification of type I NBs at 9–12, 48, and 96 hours ALH. NBs are labeled by anti-Mira in green and anti-Ase in red; thus type I NBs are both Mira- and Ase-positive and type II NBs are positive for Mira but negative for Ase. OL, optic lobe; IPC, inner proliferating center. All the quantification data in this and all the following figures are mean ± SD. *, *p*<0.05, compared with the wild type; n.s., statistically not significant.

*Drosophila* larval NSCs (called neuroblasts, or NBs) are an excellent simple model system for studying the basic, conserved biology of NSCs. In the developing fly central brain, there are two types of NBs (type I and type II). These fly NBs, especially the newly identified type II NBs (also called posterior Asense-negative [PAN] or Dorsomedial [DM] NBs), are analogous to mammalian radial glial cells, which function as NSCs in the developing mammalian brain [1]. The type II NBs produce an intermediate neural progenitor (INP) that undergoes several self-renewing divisions to amplify the number of progeny produced by each NB (Fig. 1A) [2], [3], [4]. Thus, fly INPs are comparable to the mammalian intermediate progenitor, particularly the self-renewing outer-subventricular zone radial glia-like (oRG) cells that amplify the number of progeny descended from radial glial cells as reported in human embryos and ferrets [5], [6], [7]. In each round of self-renewing divisions, individual INPs produce a ganglion mother cell (GMC), which divides once to produce two neurons. The developing fly brain, however, is mostly populated by type I NBs that give rise directly to GMCs instead of self-renewing INPs (Fig. 1A). *Drosophila* larval NBs are derived from embryonic NBs. After several rounds of self-renewing divisions, most embryonic NBs become mitotically quiescent at late embryonic stages, except for the four mushroom body NBs and a basal anterior NB. These quiescent NBs re-enter the cell cycle during 1^st^ and 2^nd^ instar larval stages and undergo repeated self-renewing divisions until early pupal stages [8], [9].

Studies of *Drosophila* NBs have identified a number of genes that regulate NB self-renewal. The majority of these genes, such as *aPKC*, *Partner of inscuteable* (*Pins*), the NuMA-related *Mushroom body defect* (*Mud*), *aurora-A*, *polo*, and *protein phosphatase 2A* (*PP2A*), are involved in asymmetric cell division (reviewed in [10], [11]. These genes ensure the proper segregation to a single daughter cell of cell fate determinants, such as Prospero (Pros), Numb, and Brat. These cell fate determinants promote cell cycle exit of GMCs or maturation of INPs [4], [12], [13]. Defects in asymmetric cell division or loss of cell fate determinants perturb the normal pattern of NB self-renewal, leading to an increased number of NBs or, conversely, differentiation. In the type II neuroblast lineage, the self-renewal potential of INPs is limited by the transcription factor Earmuff (Erm), which positively regulates *pros* expression [14]. However, the proteins that act within NBs to promote NB self-renewal remain largely unknown.

To understand how the self-renewal of NBs is regulated, we investigated the function of the *Drosophila* bHLH transcriptional repressor Deadpan (Dpn), a member of the Hairy and Enhancer-of-Split (Hes) family that is expressed in all neural precursors [15]. We reasoned that genes that are required for maintaining NBs might be specifically expressed in NBs, but not in their progeny. Dpn is specifically expressed in all the larval NBs as well as in the self-renewing INPs [3], [4], [15], making Dpn a good candidate for maintaining NB self-renewal. Our work demonstrates that Dpn is both necessary and sufficient for maintaining NB self-renewal. Furthermore, we show that Dpn and Notch function in separable pathways to suppress Ase expression in type II NBs and maintain the self-renewal of type II NBs.

## Materials and Methods

### Fly Stocks

The following fly stocks were used for *dpn* loss-of-function analyses: *dpn^1^*, *dpn^7^*, and *Df(2R)Exel^7095^*. Other fly stocks include: *FRT^42D^ dpn^7^/CyO*; *FRT^42D^ dpn^1^/CyO*; and *hs-Flpase elav-Gal4 UAS-mCD8-GFP; FRT^42D^ tub-Gal80* for generating *dpn* mutant clones; *ase-Gal4*, *insc-Gal4* (Gal4^1407^ inserted in *inscuteable* promoter), *UAS-dpn* for ectopic/over-expression of Dpn; and *Gal4^14–94^* [16] for transgene expression in type II NBs; *UAS-FLPase* and *actin-FRT-Stop-FRT-lacZ* for lineage tracing of type II NBs; *ase-Gal80* transgenic lines were generated as described below to restrict the expression of *insc-Gal4* to the type II lineages.

### Construction of ase-Gal80

The defined *ase* promoter (1.6 kb upstream of the transcription start site) together with 455 bp 5′untranslated region of the ase transcription unit [17] was amplified by PCR and subcloned into pCaSpeR4 between EcoRI and NotI. The SV40 poly(A) tail and the *Gal80* coding region were then sequentially subcloned into XbaI–StuI and NotI–XbaI sites, respectively, downstream of the *ase* promoter in pCaSpeR4 to generate *ase*-*Gal80*.

### Mosaic Analyses

*dpn* mutant MARCM [18] clones were induced by 1 hour heat shock at 38°C at 0 day or 1 day after larval hatching. Brains were dissected at various developmental stages as indicated for the examination of clonal phenotypes.

### Immunostaining and Confocal Microscopy

Larval or adult brains were dissected, fixed, and stained as described before [19]. Primary antibodies used in this study include: rabbit anti-Mira (1∶500), guinea pig anti-Ase (1∶5000), rat anti-mCD8 (Caltag, 1∶100), 1D4 mAb (Hybridoma Bank, 1∶50), mouse anti-Pros (Hybridoma Bank, 1∶20), rabbit anti-Dpn (1∶500). Secondary antibodies conjugated to Cy2, Rhodamine Red X, or Cy5 (Jackson ImmunoResearch, West Grove, PA, USA) were used at 1∶100, 1∶500, or 1∶50, respectively. Alexa Fluor 488 Phalloidin (Invitrogen Carlsbad, CA, USA) was used at 1∶40. Images were taken with Leica SP2 and SP5 confocal microscope and processed with Adobe Photoshop. Two-tailed t-tests were used for statistical analyses.

## Results

### Loss of *dpn* Results in a Complete Absence of Type II NBs and a Reduction of Type I NBs in Late 3^rd^ Instar Larval Brains

There are approximately 100 NBs in the central brain of each *Drosophila* larval brain lobe. The majority of these NBs are specified during embryonic stages. After a period of quiescence during late embryonic and early larval stages, these NBs are reactivated and proliferate until around 1 day after pupal formation (APF), at which point they undergo terminal divisions [8]. An exception to this pattern are the mushroom body NBs, which proliferate continuously from embryonic stages until the end of pupal stages [9]. We predict that the loss of any gene necessary for NB maintenance will result in a premature disappearance of NBs, and thus fewer NBs during larval stages. To examine whether Dpn is required for maintaining NB self-renewal, we first compared the number of type I and type II NBs in wild type and *dpn* mutant brains at the late 3^rd^ instar larval stage (4 days after larval hatching [ALH]) using Miranda (Mira), which marks all larval NBs, and Asense (Ase), which is expressed in type I but not type II NBs. Three *dpn* alleles, including the null allele *dpn^1^*, the dominant negative allele *dpn^7^*, and the deficiency line *Df(2R)Exel^7095^* that removes the entire *dpn* gene, were used to examine *dpn* mutant phenotypes (note that the *dpn^7^* allele produces a dominant-negative Dpn protein that is still recognized by the anti-Dpn antibody). Each brain lobe in a wild type 3^rd^ instar larva normally contains eight type II NBs on the dorsal side (Fig. 1B) and approximately 90 type I NBs located on both dorsal and ventral sides (Fig. 1D). Strikingly, type II NBs were completely absent in *dpn^1^/dpn^7^* mutant brains (Fig. 1C). The number of type I NBs was also significantly reduced to 45–65 type I NBs per brain lobe in *dpn^1^/dpn^7^* mutants (Fig. 1E, F). Similar results were observed in *dpn^1^*/*Df(2R)Exel^7095^* and *dpn^7^/Df(2R)Exel^7095^* transheterozygous larval brains (data not shown). These results are consistent with the results recently reported by San-Juan and Baonza (2011), and Zacharious et al. ([20], [21].

### A Reduced Number of Type II NBs Exist in *dpn* Mutant 1^st^ Instar Larval Brains and Ectopically Express Ase and Pros

The complete loss of type II NBs in 3^rd^ instar *dpn* mutant brains could reflect (1) a premature disappearance of NBs during embryonic or early larval stages, (2) an inability to generate NBs during embryogenesis, (3) a transformation of type II NBs into type I NBs, or (4) a failure to exit quiescence at early larval stages. One way to distinguish these possibilities is to remove Dpn after type II NBs are formed by generating *dpn* mutant clones. Although Dpn is absent in *dpn^1^* whole mutant animals (Fig. S1A–B’), Dpn protein perdures in *dpn^1^* clones. When *dpn^1^* clones were induced at 1 day ALH and examined 2 days later, Dpn was still detected in both type I and type II NBs, albeit at much lower levels than in neighboring *dpn^1^* heterozygous NB lineages (Fig. S1C, C’, E, E’). Only 4 days after clone induction was Dpn reduced to undetectable levels in *dpn^1^* mutant clones (Fig. S1D, D’, F, F’). The perdurance of Dpn in *dpn* mutant clones made it difficult to unambiguiously determine the role of Dpn in larval NBs (except for MB NBs, see below) by clonal analyses.

**Figure 2:**
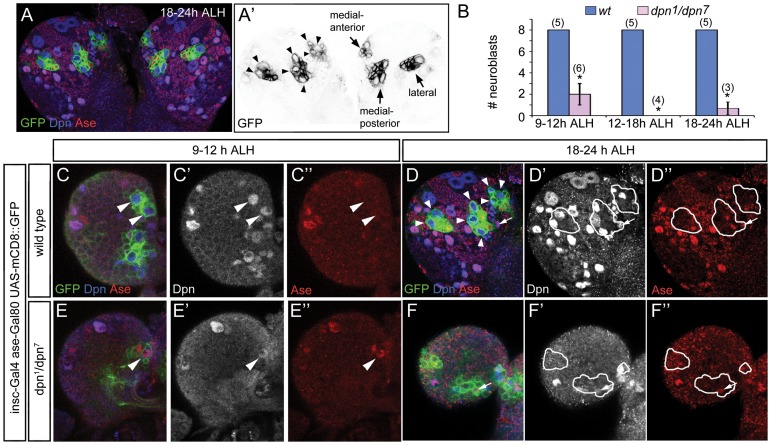
*dpn^1^*/*dpn^7^* 1^st^ instar larval brains contained a reduced number of type II NBs, which ectopically express Ase. Type II NB lineages are labeled by *UAS-mCD8-GFP* driven by *insc-Gal4 ase-Gal80* in green. (A, A’) In wild type brains at 18–24 hours ALH (late 1^st^ instar), three clusters of cells in each lobe are labeled by *UAS-mCD8-GFP* driven by *insc-Gal4 ase-Gal80*. Two clusters containing 3 type II NBs each are located medially, one anterior and one posterior. A third cluster containing 2 type II NBs is located laterally. (B) Quantification of the number of type II NBs in wild type and *dpn^1^*/*dpn^7^* brain lobes at the 1^st^ instar larval stage. Numbers in parentheses indicate the sample size. *, *p*<0.05. (C–D”) In wild type brain lobes, type II NBs (arrowheads) can be distinguished as early as 9–12 hours ALH (C’–C”), and their progeny, including immature and mature INPs (e.g. arrows), are more clearly visible at 18–24 hours ALH (D–D”). Note that NBs are typically at least twice the size of INPs and neurons. (E–F”) In *dpn^1^*/*dpn^7^* brain lobes at 9–12 hours ALH, there is only an occasional NB (arrowhead), which ectopically expresses Ase (E”); at 18–24 hours ALH (F–F”), there are a few remaining small Ase^+^ Dpn^+^ cells (e.g. arrows) within the type II NB clusters that are likely remaining mature INPs.

To investigate when type II NBs are lost in *dpn* mutant brains, we first characterized the development of type II NB lineages in wild type 1^st^ instar larvae and looked for type II NBs in *dpn^1^/dpn^7^* mutants at the same stage. To specifically label type II NBs and their progeny, we used *UAS-mCD8-GFP* driven by a combination of *inscuteable* (*insc*)-*Gal4* [22] and *asense (ase)-Gal80*. *Insc*-*Gal4* is active in all NBs whereas *ase-Gal80* is expressed only in type I NBs. Therefore, *insc-Gal4* in combination with *ase-Gal80* restricts the expression of *mCD8-GFP* to type II NB lineages (Fig. 2A, also see Fig. S2A, A’). Using this approach, the earliest time point at which we were able to definitively identify all 8 type II NBs in wild type brains was 9–12 hours ALH, when the type II NBs have likely just exited quiescence (Fig. 2B–C”). In wild type 1^st^ instar larvae, type II NBs and their progeny are clustered into 3 groups in each brain lobe: two groups, comprised of 3 NBs each, are positioned on the medial side of the brain and 2 NBs are clustered on the lateral side (Fig. 2A’). Consistent with the notion that type II NBs were just emerging from quiescence at this time, there were relatively few INPs and GMCs at 9–12 hours ALH (Fig. 2C–C”). At 18–24 hours ALH, each brain lobe contained increased numbers of type II NB progeny, including mature INPs and GMCs (Fig. 2D–D”).

In *dpn^1^/dpn^7^* brain lobes at 9–12 hours ALH, we observed 3 clusters of mCD8-GFP^+^ cells in each brain lobe, but only identified on average 2 mCD8-GFP-labeled NBs per brain lobe (Fig. 2B, E–E”). After 9–12 hours ALH, we rarely observed type II NBs in the mCD8-GFP^+^ clusters in *dpn^1^*/*dpn^7^* brains, except for some small Ase^+^ Dpn^+^ cells resembling INPs (Fig. 2B, F, F’, also see Fig. S2B, B’). Interestingly, unlike normal type II NBs, which do not express Ase and Pros, the mCD8-GFP-labeled *dpn* mutant NBs observed at 9–12 hours ALH ectopically expressed Ase and nuclear Pros, making type II NBs appear similar to type I NBs at the same stage (Fig. 2E–E’, Fig. S3). To investigate whether type II NBs are generated and specified correctly, we examined type II NBs in wild type and *dpn* mutant brains at embryonic stage 14/15 (10.5–13 hours after egg laying [AEL]). Interestingly, unlike larval brains, which contain only 8 Dpn^+^ Ase^-^ NBs per hemisphere, embryonic brains contain 33.2±4 (n = 6) Dpn^+^Ase^-^ NBs per hemisphere at stage 14/15 (Fig. S4A–A”). A similar number (32.3±4.8, n = 6) of Dpn^+^Ase^-^ NBs were also observed in *dpn* mutant brains at the same stage (Fig. S4B–B”). Although it is not clear which of these embryonic Dpn^+^Ase^-^ NBs become larval type II NBs, these results indicate that the loss of Dpn likely does not affect the generation and specification of type II NBs at embryonic stages. The ectopic expression of Ase and Pros in *dpn* mutant type II NBs during early larval stages suggests that Dpn may be required for maintaining their proper identity of type II NBs only after they exit quiescence, but not earlier during embryonic stages. In the absence of Dpn, type II NBs are transformed into type I-like NBs after they exit quiescence and may eventually lose the expression of mCD8-GFP driven by *insc-Gal4 ase-Gal80* due to the activation of the *ase* promoter and subsequent expression of Gal80. However, since no ectopic GFP^-^ type I NBs were found at locations where type II NBs reside (Fig. 2F, F’, F”), it is likely that these transformed *dpn* mutant type II NBs were lost prematurely due to a defect in maintaining self-renewal soon after they exit quiescence.

### Mushroom Body NBs Lacking Dpn are Prematurely Lost, Resulting in Truncated Lineages

**Figure 3:**
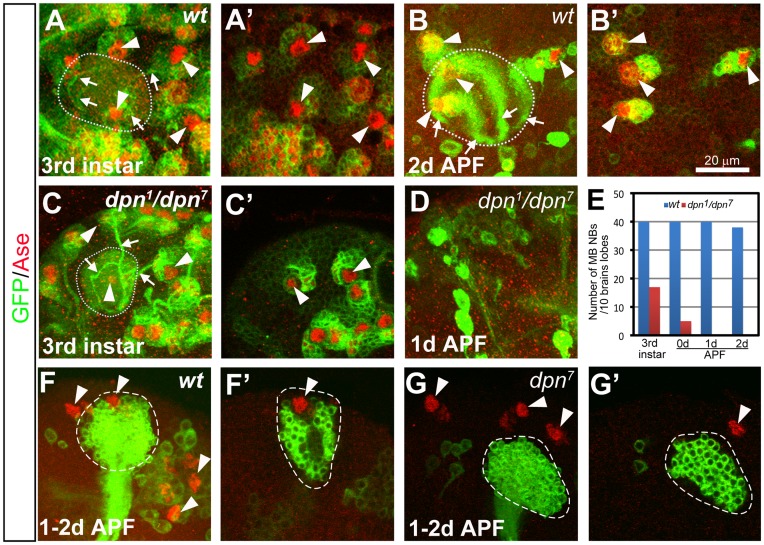
Progressive loss of mushroom body (MB) NBs in *dpn* mutants. (A–B’) A wild type MB has four NBs (arrowheads) at the 3^rd^ instar larval stage (A–A’) and 2 days APF (B–B’). (C–D) A *dpn* mutant brain still has three MB NBs at the 3^rd^ instar larval stage (arrowheads in (C). Only two of them were shown in a single focal plane in (C’)), but none at 1day APF (D). (A, B, C, and D) are composite confocal images and (A’, B’, and C’) are single focal slices of (A, B, and C) respectively. NBs are labeled by mCD8-GFP expression (green) driven by *insc*-*Gal4* and Ase staining (red). Dotted circles outline the MB dendritic calyces. Arrowheads indicate the MB NBs. Arrows point to the bundles of primary neurites from the newly born neurons. (E) Quantification of the total number of MB NBs in 10 wild type or *dpn* mutant brain lobes at different developmental stages. (F–F’) A wild type MB NB clone labeled with mCD8-GFP in green still contains a NB at 1–2d APF. Total 4 MB NBs (arrowheads) can be observed in a brain lobe at 1–2d APF, including the one that is associated with the MB NB clone. (G–G’) A *dpn^7^* mutant MB NB clone has no associated NB at 1–2d APF, resulting in only 3 MB NBs (arrowheads) in total in one brain lobe. (F and G) are composite confocal images and (F’ and G’) are single-focal planes.

To investigate whether the reduction of type I NBs in 3^rd^ instar *dpn* mutant brains is due to either a premature loss or a defect in NB formation, we next examined at different developmental stages the well-characterized type I NB lineages that produce the two bilaterally symmetric mushroom bodies (MBs) in the adult fly brain. A single MB is derived from four NBs, each of which divides repeatedly from late embryonic to late pupal stages to sequentially generate three distinct types of MB neurons (γ, α’/β’, and α/β) (Fig. S5A, B) [19]. To analyze the MB lineage, we used *insc-Gal4*, which labels all larval NBs, to drive the expression of mCD8-GFP. *UAS-mCD8-GFP* driven by *Insc-Gal4* labels not only MB NBs, but also the newly born MB neurons and their neurites, which form a distinctive structure. By tracing the neurite bundles from the MB dendritic calyces back to their cell bodies it is possible to identify MB NBs (Fig. 3A, B). In wild type animals, each brain lobe has 4 MB NBs until 2 days APF (Fig. 3A–B’, E). However, in *dpn^1^*/*dpn^7^* mutants, MB NBs were progressively lost. At the late 3^rd^ instar larval stage, nearly half of MB NBs were missing in *dpn* mutant brains (Fig. 3C, C’, E) and by the start of pupariation, 90% of MB NBs were lost (Fig. 3E). By 1 day APF, no MB NBs were found (Fig. 3D, E). Premature progressive loss of *dpn* mutant MB NBs suggests that Dpn is required to maintain MB NB self-renewal.

In order to assess whether Dpn functions cell-autonomously to maintain MB NB self-renewal, we next examined *dpn* loss-of-function phenotypes in MB NB clones that were generated right after larval hatching. The MB NBs keep dividing till the end of pupal stage and produce the longest lineages in the fly brain. Given that Dpn perdures in *dpn* mutant clones for at least 2 days after clone induction, we examined the phenotype in MB NB clones at 1–2d APF, which is about 5–6 days after clone induction. At 1–2d APF, wild type MB NB clones always contained a NB (n = 10) (Fig.3F–F’). In contrast, most (7/8) *dpn^7^* mutant MB NB clones had lost their NBs at 1–2d APF (Fig. 3G–G’). As a result, *dpn* mutant MB NBs failed to produce late-born MB neurons, resulting in truncated MB lineages that contained fewer MB neurons in adults (Fig. S5C–F). The premature loss of the MB NBs in *dpn* mutant clones suggests that Dpn functions cell-autonomously to maintain MB NB self-renewal.

### The Lose of Non-MB Type I NBs Occurs Mainly within 48 Hours ALH in *dpn* Mutant Brains

To determine whether the loss of other type I NBs in *dpn* mutant larval brains is similarly due to a defect in maintaining NB self-renewal, we counted the number of type I NBs in wild type and *dpn* mutant larval brains at different developmental stages. In *Drosophila* larval brains, NBs start to exit quiescence after 8 hours ALH. By the end of the second instar larval stage, the majority of NBs have exited quiescence and remain proliferative until 1d APF [9]. We compared the number of type I NBs in wild type and *dpn* mutant larval brains at 9–12, 48, and 96 hours ALH. At 9–12 hours ALH, although most NBs were still quiescent and very small, we were able to identify 94±6.7 type I NBs in wild type brains based on Dpn and Ase staining. The number of type I NBs remained similar at 48 and 96 hour ALH in wild type animals (Fig. 1F, data not shown). In *dpn* mutant larval brains, the number of type I NBs was close to that in wild type animals at 9–12 hours ALH (87.8±6.7), but was reduced to 64.2±7.1 at 48 hours ALH and remained stable from 48 hours ALH to 96 hours ALH (Fig. 1F). Therefore, the loss of non-MB type I NBs in *dpn* mutant brains is also due to a defect in maintaining NB self-renewal, but this loss of NBs mainly occurred within 48 hours ALH.

### Pros Likely Mediates the Premature Loss of *dpn* Mutant Type I NBs

**Figure 4:**
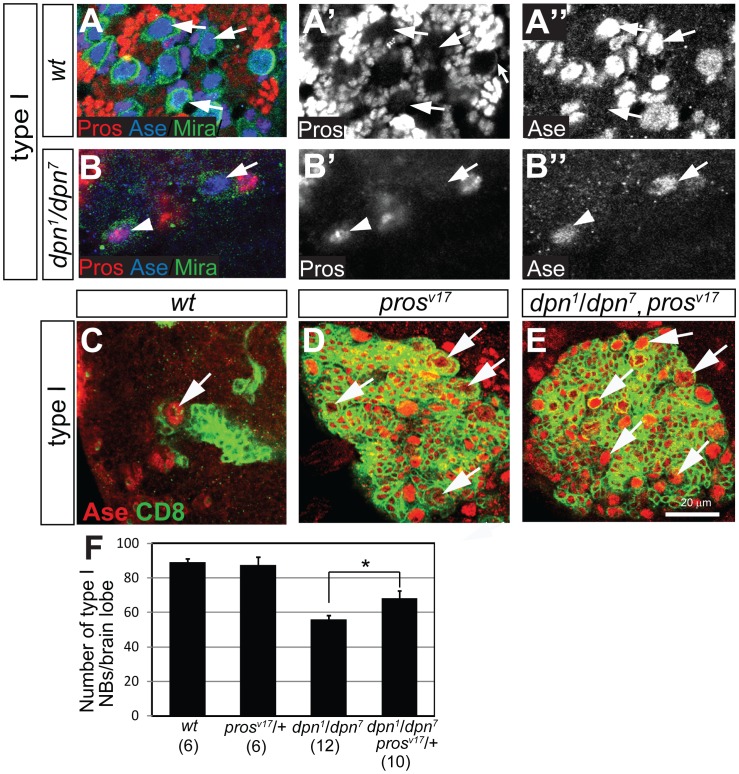
Pros mediates premature terminal division of *dpn* mutant NBs. (A–A’’) Wild type NBs (large arrows) do not accumulate nuclear Pros at the 3^rd^ instar larval stage. Instead, only cortical Pros is detected (the small arrow in (A’)). (B–B’’) Nuclear Pros is detected in a *dpn* mutant type I NB (arrowhead) at the 3^rd^ instar larval stage, while a neighboring NB (arrow) remains negative for nuclear Pros. NBs in (A–B) are labeled with Mira staining in green. (C–E) Wild type (C), *pros^v17^* (D), and *dpn pros^v17^* double mutant (E) type I NB clones at the 3^rd^ instar larval stage. Clones were labeled with mCD8-GFP and stained for Ase in red. Arrows point to NBs in the clones. (F) The number of *dpn* mutant type I NBs at late 3^rd^ instar larval brains is partially rescued in *pros^v17^* heterozygous mutant background, whereas the number of type I NBs in *pros^v17^* heterozygous larval brains is similar to that in wild type brains.

The premature loss of NBs in *dpn* mutant brains could be due to either apoptosis or precocious terminal division. Apoptosis is unlikely the cause of NB loss since activated caspase was not detected in *dpn* mutant NBs and the ectopic expression of the caspase inhibitor p35 failed to rescue NB loss (data not shown). In developing fly brains, the majority of NBs (except the MB NBs) undergo a terminal division around 1 day APF [9]. Previous work revealed that the terminal division of most central brain NBs is correlated with the nuclear accumulation of the transcription factor Pros, which promotes cell cycle exit [12], [13]. To test the possibility that Pros prematurely accumulates in the nuclei of *dpn* mutant NBs and causes their terminal divisions, we first examined whether Pros precociously accumulates in the nuclei of *dpn* mutant type I NBs at the 3^rd^ instar larval stage. In type I NBs of wild type 3^rd^ instar larvae, only cortical Pros was detected (Fig. 4A–A”). In contrast, Pros was present in the nuclei of multiple type I NBs in 3^rd^ instar *dpn^1^*/*dpn* ^7^ brains (Fig. 4B–B”), suggesting that *dpn* mutant type I NBs undergo Pros-mediated premature terminal division.

To further determine whether Pros mediates the premature terminal division of *dpn* mutant NBs, we tested (1) whether the loss of Pros would prevent the premature loss of *dpn* mutant NBs by generating *pros* loss-of-function (*pros^v17^*) clones in *dpn^1^*/*dpn* ^7^ mutant brains, and (2) whether reducing Pros amounts could partially rescue the loss of type I NBs in *dpn* mutant larval brains. Consistent with previous reports [4], [23], *pros^v17^* type I NB clones encompassed multiple Ase^+^ NBs (Fig. 4D, compared with the wild type clone in Fig. 4C). *pros^v17^* mutant type I NB clones generated in *dpn^1^*/*dpn* ^7^ brains had a similar number of NBs (Fig. 4E) as those generated in wild type brains at the 3^rd^ larval stage (13.7±2.1 NBs/clone in *dpn^1^*/*dpn* ^7^ brains, n = 3; 14.3±3.8 of NBs/clone in wild type brains, n = 3). Furthermore, these *pros dpn* double mutant type I NBs were maintained through adulthood like *pros* mutant type I NBs (Fig. S6A–B), whereas a wild type adult brain does not contain any NBs (see below). Consistently, when *pros* expression was reduced by removing one wild type copy of *pros*, the number of *dpn* mutant type I NBs increased by about 15% (Fig. 4F). These data suggest that the premature terminal division of *dpn* mutant type I NBs is likely mediated by Pros. However, we did not find any *pros^v17^* mutant clones with Ase^-^ type II NBs in *dpn^1^*/*dpn* ^7^ brains (data not shown). Since Ase is ectopically expressed in *dpn* mutant type II NBs as early as the 1^st^ instar larval stage (Fig. 2E–E”), it is possible that Ase remained mis-expressed in *pros^v17^* type II NB clones in *dpn^1^*/*dpn* ^7^ mutant 3^rd^ instar larvae. The ectopic expression of Ase makes it difficult to conclude whether *dpn* mutant type II NBs are maintained in the absence of Pros.

### Ectopic/over-expression of Dpn Leads to Over-proliferation and Failure to Properly Terminate Self-renewal in Both Type I and Type II NBs

**Figure 5:**
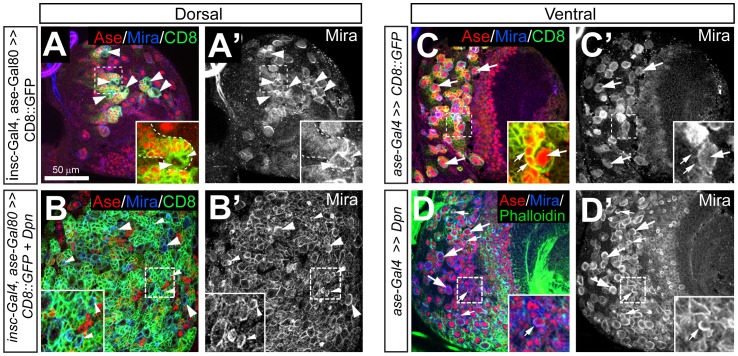
Ectopic/over-expression of Dpn results in an increased number of NBs and the ectopic self-renewal of immature INPs and GMCs in larval brains. (A–A’) *insc-Gal4 ase-Gal80* drives mCD8-GFP expression in type II NB lineages in a wild type brain. Insets show that mCD8-GFP is expressed in all the INPs, including immature INPs (small arrowheads), in addition to NBs (large arrowheads) in the type II lineages. Only 6 NBs (large arrowheads) are shown in a single focal slice. (B–B’) Dpn expression driven by *insc-Gal4 ase-Gal80* in the type II lineages leads to numerous type II NBs (e.g. large arrowheads) and Ase^-^ immature INPs (e.g. small arrowheads). Insets show that Mira forms crescents in some immature INPs (small arrowheads) in the region highlighted by dashed squares. (C–C’) *ase-Gal4* drives the expression of mCD8-GFP in the type I NB lineages. Insets: CD8-GFP is expressed in both NBs (large arrows) and neighboring GMCs (small arrows). (D–D’) Dpn expression in type I NB lineages driven by *ase-Gal4* (D–D’) results in an increase in the number of type I NBs (e.g. large arrows) as well as many small Mira^+^ Ase^+^ GMCs (e.g. small arrows). Insets show that Mira forms a crescent in a small Ase^+^ GMC in the region highlighted by dashed squares.

Having found that *dpn* is necessary for maintaining NB self-renewal, we next asked whether Dpn is sufficient to promote NB self-renewal by testing whether the ectopic expression of Dpn in non-self-renewing cells such as immature INPs or GMCs results in ectopic self-renewal, and whether over-expressing Dpn in NBs results in abnormally prolonged self-renewal. If Dpn is sufficient to promote self-renewal, the ectopic expression of Dpn in non-dividing immature INPs and terminally dividing GMCs should lead to ectopic proliferation and increased numbers of immature INPs and GMCs in type II and type I NB lineages. The ectopically proliferating immature INPs and GMCs may even adopt a NB-like fate, leading to increased numbers of type II and type I NBs. Furthermore, Dpn ectopic/over-expression may prolong the maintenance of NBs into adulthood. Since no immature INP- or GMC-specific Gal4 lines are available, we used *insc*-*Gal4 ase-Gal80* for Dpn ectopic/over-expression in type II NB lineages, and *ase-Gal4* [24] or *insc*-*Gal4* for Dpn expression in type I NB lineages. In type II lineages, *UAS-CD8-GFP* driven by *insc-Gal4 ase-Gal80* was expressed not only in the NBs but also in INPs and GMCs (Fig. 5A). Similarly, in the type I NB lineage, *UAS-CD8-GFP* driven by *ase-Gal4* or *insc*-*Gal4* was expressed in both NBs and GMCs (Fig. 5C and data not shown). Therefore, these drivers allowed us to over/ectopically express Dpn in NBs as well as immature INPs and GMCs.

**Figure 6:**
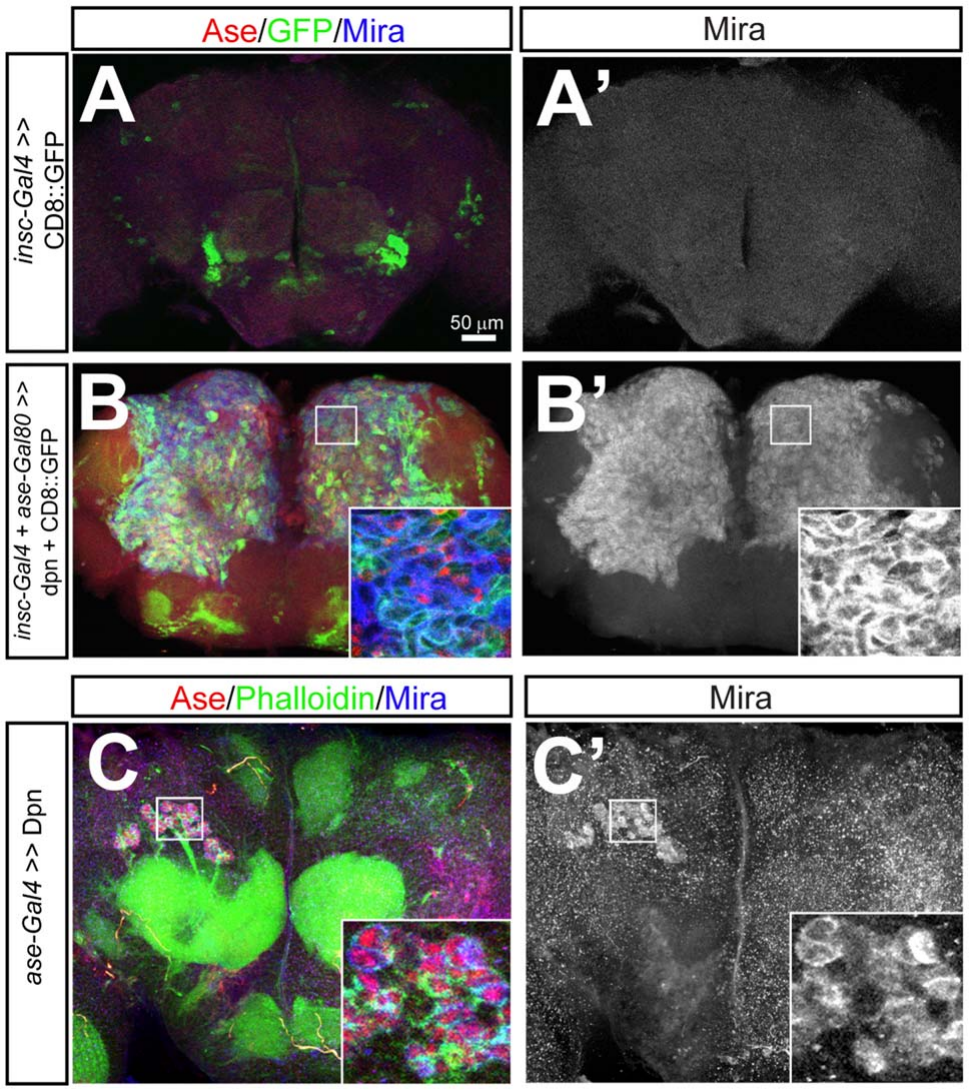
Ectopic NBs persist in adult brains after Dpn is over-expressed. ( A–A’) Wild type adult brains do not have any NBs, as indicated by the absence of Mira^+^ cells (A’). **(**B–B’) Ectopic**/**over-expression of Dpn in the type II NB lineages using *insc*-*Gal4 ase-Gal80* leads to numerous Ase^-^ type II NBs and immature INPs in adult brains. (C–C’) Ectopic**/**over-expression of Dpn in type I NBs using *ase*-*Gal4* results in ectopic Ase^+^ type I NBs in adult brains. Mira staining (blue) labels NBs. Insets in (B) and (C) show enlarged views of the highlighted areas.

We first examined how Dpn ectopic/over-expression affected type II NB lineage development. Strikingly, ectopic/over-expression of Dpn resulted in a massive increase in the number of type II NBs (131±15 type II NBs per brain lobe, Fig. 5B, B’) and numerous small Ase^-^ immature INPs (Fig. 5B) in 3^rd^ instar larval brains, compared to 8 type II NBs and 2–3 immature INPs in each type II NB lineage in wild type brains (Fig. 5A). Moreover, the ectopic type II NBs and immature INPs resulting from Dpn ectopic/over-expression even existed in adult brains (Fig. 6B, B’), which normally do not have any NBs or INPs (Fig. 6A, A’) [9], [13], [25]. Similarly, when Dpn was ectopically/over-expressed in type II NB clones using *insc-Gal4*, the clones were much larger and contained more NBs and immature INPs than wild type clones (11.8±3.1 type II NBs and 94.6±13 immature INPs in clones over-expressing Dpn, n = 5; control type II NB clones had only a single NB and 2.3±0.5 immature INPs, n = 6) (see Fig. S7A, B). The ratio of immature INPs to NBs also increased from 2∶1 to 8∶1, suggesting that Dpn over-expression mainly promotes the over-proliferation of immature INPs. As a result, clones over-expressing Dpn contained relatively fewer mature INPs and neurons per NB than control clones (4.6±1.4 mature INPs/NB and 7.9±1.5neurons/NB in clones over-expressing Dpn, n = 5; 19.3±8 mature INPs/NB and 45.4±10.7 neurons/NB in wild type clones, n = 6), indicating that the proliferating immature INPs mainly produced immature INPs and/or type II NBs rather than giving rise to mature INPs and neurons.

To confirm that immature INPs indeed undergo ectopic self-renewing divisions in response to Dpn ectopic/over-expression, we examined the expression of a mitotic marker phospho-histone H3 (p-H3) and Mira in these immature INPs. Immature INPs normally do not divide and are negative for p-H3 (Fig. S8A, A’). However, in larval brains over/ectopically expressing Dpn, many immature INPs were ectopically labeled by p-H3 (Fig. S8B, B’), indicating that they were actively dividing. Moreover, Mira formed a crescent in some of these mitotic immature INPs (insets in Fig. 5B, B’, compared to the even distribution of Mira on the membrane of wild type immature INPs as shown in insets in Fig. 5A, A’). This asymmetric localization pattern is reminiscent of Mira distribution in NBs (Fig. 5B, B’), indicating that these immature INPs divided asymmetrically. The ectopic self-renewing division of immature INPs and the prolonged existence of type II NBs in adult brains resulting from Dpn ectopic/over-expression provide strong evidence that Dpn is sufficient to promote NB self-renewal.

Similar to the overproliferation phenotypes observed in type II NB lineages, ectopic/over-expression of Dpn driven by *ase-Gal4* or *insc-Gal4* (data not shown) in type I NB lineages also resulted in an increased number of NBs and GMCs (Fig. 5D–D’, compared to Fig. 5C, C’; also see Fig. S7C–E’), some of which even persisted in adult brains (Fig. 6C, C’). Furthermore, Mira also formed a crescent in some GMCs (insets in Fig. 5D, D’), as in the case of immature INPs upon Dpn over-expression. These observations suggest that ectopic/over-expression of Dpn is sufficient to cause terminally dividing GMCs to undergo self-renewing asymmetric divisions. Some of these ectopically self-renewing GMCs may eventually enlarge and become NBs, resulting in an increased number of type I NBs. However, compared to the over-proliferation phenotypes observed in type II NB lineages, the over-proliferation of type I NBs and GMCs in response to ectopic/over-expression of Dpn in type I NB lineages is milder. Dpn ectopic/over-expression in type I NB clones only increased the number of type I NBs by 2 fold and GMCs by 3 fold (1.7±1.5 NBs and 18.2±19.7 GMCs in clones over-expressing Dpn, n = 11; 1 NB and 5.7±1.7 GMCs in wild type clones, n = 11), whereas Dpn ectopic/over-expression in type II NB clones resulted in a more than 10-fold increase in the number of type II NBs and an over 50-fold increase in the number of immature INPs. Furthermore, ectopic type I NBs resulting from Dpn ectopic/over-expression only persisted in half of adult brain examined (n = 15) (Fig. 6C, C’), whereas ectopic type II NBs existed in every adult brains (n = 15) (Fig. 6B, B’).

Taken together, Dpn ectopic/over-expression phenotypes in both larval and adult brains suggest that Dpn is sufficient to promote the self-renewal of both NB types, rather than just type II NBs as reported by San-Juan and Baonza [21]. However, type II NB lineages are more susceptible than type I NB lineages to the increase in Dpn expression levels.

### Dpn does not Function Downstream of Notch in Type II NBs

**Figure 7:**
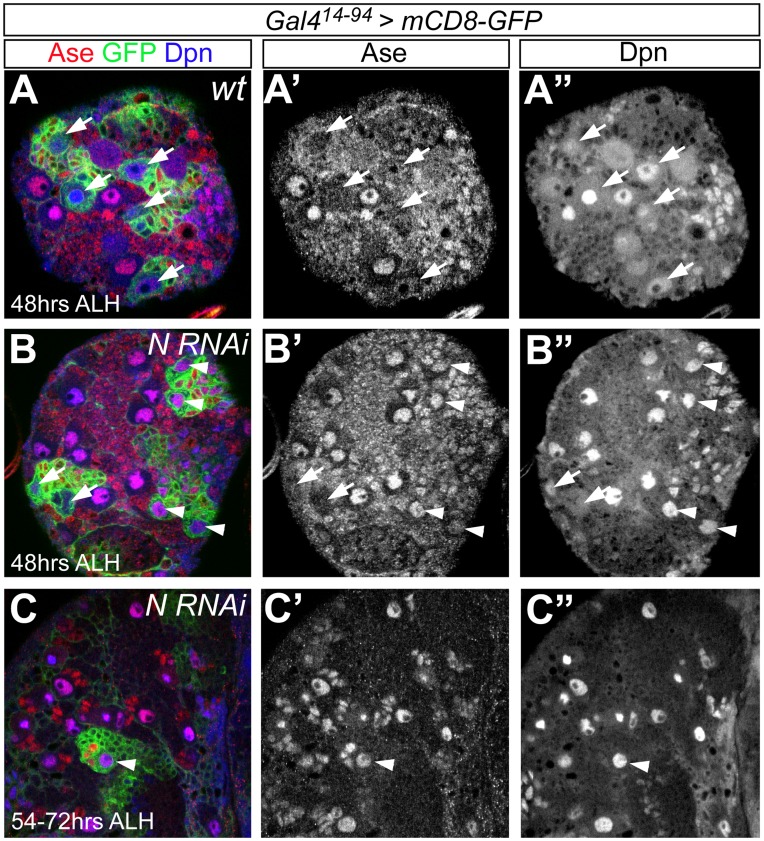
Knockdown of Notch results in the ectopic expression of Ase in type II NBs and the premature loss of type II NBs. (A–A”) mCD8-GFP driven by *Gal4^14–94^* specifically labels type II NBs (arrows) at 48 hours ALH. (B–C”) Knockdown of Notch by *Notch* RNAi driven by *Gal4^14–94^* results in the ectopic expression of Ase in type II NBs (arrowheads) and the gradual loss of type II NBs. Arrows point to the type II NBs that remain Ase^-^. Note that Dpn expression in type II NBs ectopically expressing Ase (arrowheads) remains similar to that in neighboring type I NBs or wild type type II NBs (A”) after Notch is knocked down.

*Drosophila* Hes family proteins, such as Hairy and Enhancer-of-split, are known to mediate Notch signaling [26]. Upon activation of the Notch receptor, the intracellular domain of Notch (N^IC^) translocates to the nucleus and activates the transcription of Hes family proteins. In *Drosophila*, Notch is expressed in larval NBs [27]. Decreasing Notch activity, either by RNAi knockdown or by expressing Numb, largely eliminates type II NBs, whereas increasing Notch activity by expressing the intracellular domain of Notch (N^IC^) results in a dramatic increase in NB numbers, particularly the type II NBs [4], [28], [29]. These phenotypes are similar to those caused by manipulating Dpn levels in type II NBs. Therefore, we asked whether Dpn functions downstream of Notch in type II NBs. To address this question, we first examined Dpn expression at different developmental stages when Notch is knocked down in type II NBs using *Notch* RNAi driven by *Gal4^14–94^*, which is expressed in type II NBs and INPs but not in type I NBs lineages (Fig. 7A-A”, Fig. S9A-A”) [16]. Knockdown of Notch leads to the progressive loss of type II NBs labeled by mCD-GFP driven by *Gal4^14–94^*. At 48 hours ALH, we observed 6.3±1.1 (n = 7) type II NBs per brain lobe (Fig. 7B–B”). The number of type II NBs was further reduced to 1.8±0.8 per brain lobe (n = 6) (Fig. 7C–C”) at 54–72 hours ALH. At 96 hours ALH, no type II NBs were observed (n = 8) (Fig. S8B–B”). However, the amount of Dpn in the remaining type II NBs at 48 or 54–72 hours ALH stayed similar to neighboring type I NBs, suggesting that the knockdown of Notch does not affect the expression of Dpn in type II NBs. Interestingly, we found that the knockdown of Notch led to the ectopic expression of the type I NB marker Ase in type II NBs (Fig. 7B–C”). Furthermore, Dpn^+^Ase^+^ INPs were largely eliminated at 54–72 hours ALH in the remaining type II NBs lineages. Instead, only a few Ase+ GMC-like cells were observed beside the remaining type II NBs, making the type II NB lineages appear as type I-like NB lineages (Fig. 7C–C”). The ectopic expression of Ase in type II NBs led us to wonder whether the progressive loss of type II NBs resulting from the knockdown of Notch is due to a transformation of type II NBs into type I NBs. We next performed a lineage tracing experiment using *UAS-FLPase* and *actin-FRT-Stop-FRT-lacZ*. The expression of *FLPase* driven by *GAL4^14–94^* in type II NBs and their progeny will lead to the excision of the stop codon in *actin-FRT-Stop-FRT-lacZ* and subsequent expression of *lacZ* under the control of the *actin* promoter (Fig. S9A–A”). Since the *actin* promoter is constitutively active, this approach allows us to label type II NB lineages with *lacZ* independent of type II NB-specific driver at late larval stages. Using this approach, we labeled the progeny derived from type II NBs with *lacZ* at 96 hours ALH when Notch was knocked down. However, we did not observe any NBs expressing *lacZ*, suggesting that knocking down Notch results in a defect in the maintenance of type II NB self-renewal. Therefore, Notch is required for maintaining the self-renewal as well as the identity of type II NBs throughout larval development, but does not regulate the expression of Dpn in type II NBs.

**Figure 8:**
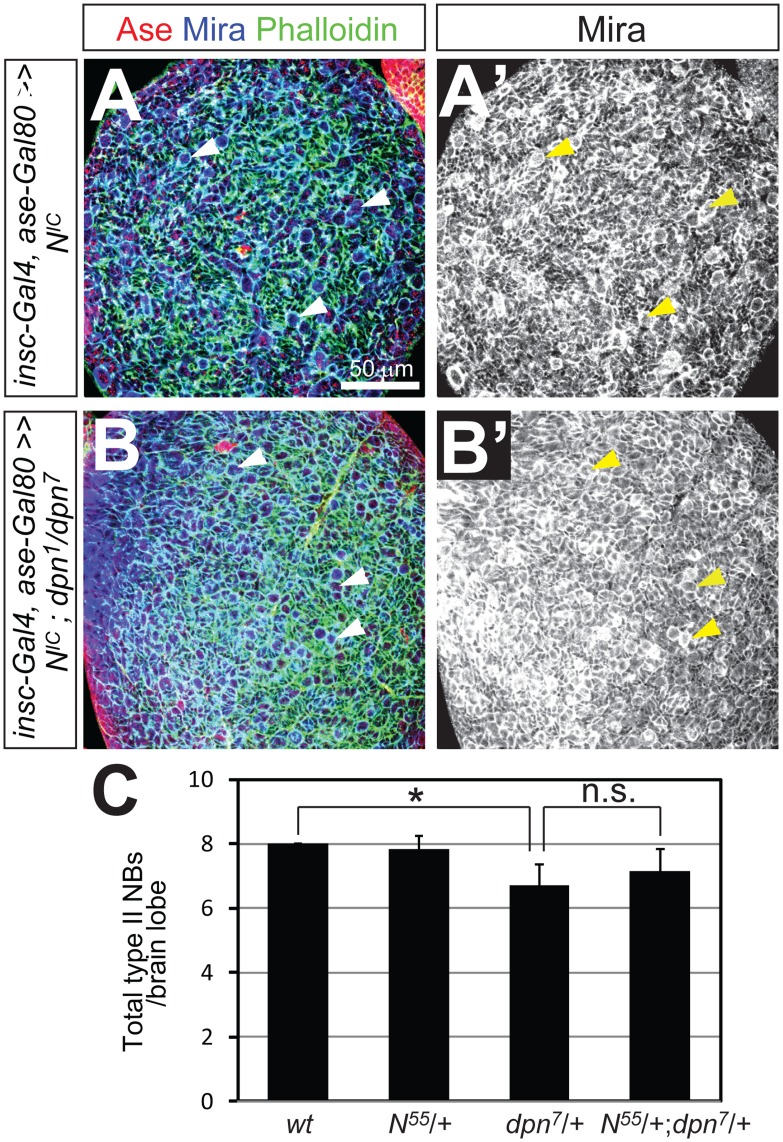
Dpn does not function downstream of Notch in maintaining type II NB self-renewal. (A–A’) Expression of the intracellular domain of Notch (N^IC^) driven by *insc-Gal4 ase-Gal80* in wild type type II NB lineages leads to an over-proliferation of type II NBs (e.g. arrowheads) and numerous smaller Ase^-^ immature INPs. (B–B’) Expression of N^IC^ driven by *insc-Gal4 ase-Gal80* in *dpn* mutant type II NB lineages leads to a similar over-proliferation of type II NBs (arrowheads) and immature INPs. (C) Quantification of the number of type II NBs in 3^rd^ instar larval brains with indicated genotypes (n = 6 in each group). *, *p*<0.05; n.s., stastically not significant.

To further explore the possibility that Dpn functions independently of Notch in type II NBs, we also performed the following two genetic interaction tests. First, we examined whether loss of Dpn inhibits the over-proliferation of type II NBs caused by over-expression of activated Notch. Over-expression of activated Notch, the intracellular domain of Notch (N^IC^), or loss of Numb leads to the overproliferation of both type I and type II NBs [4], [28], [29]. We found that over-expressing N^IC^ using *insc-Gal4 ase-Gal80* resulted in similar over-proliferation of type II NBs in wild type and *dpn* mutant larval brains (Fig. 8A–B’). Second, we examined whether lowering Notch expression enhances the loss of type II NBs in *dpn^7^* heterozygous larval brains. *dpn^7^* heterozygous mutant larvae contained a slightly reduced number of type II NBs (about 6 per brain lobe) (Fig. 8C), thus providing a sensitized genetic background. However, we found no further reduction of the number of type II NBs in *Notch dpn^7^* trans-heterozygous mutant brains. These genetic data further suggest that Dpn and Notch function in separable pathways to regulate type II NB self-renewal and identity.

## Discussion

Dpn was initially identified as a pan-neural protein about two decades ago [15] and has been widely used as a NB marker. However, the function of Dpn in NBs has been elusive. In this study, we provided evidence that Dpn plays an important role in maintaining NB self-renewal. In type II NBs, in addition to maintaining the self-renewal, Dpn is also required to suppress Ase expression when these NB exit quiescence. Furthermore, we demonstrate that Notch and Dpn may function independently in larval NBs. While both Dpn and Notch are required for maintaining the identity and self-renewal of type II NBs, knockdown of Notch does not affect the expression of Dpn in type II NBs.

### Dpn maintains the Self-renewal of Larval NBs

In a developing nervous system, NSCs must be maintained when they divide in order to generate the complete array of neurons and glia that form a functional neuronal circuit. Current studies are focused on determining how NSC self-renewal is maintained, as well as mechanisms governing NSC terminal differentiation (reviewed in [30]). Our findings that *dpn* mutant MB NBs as well as other type I NBs are premature progressive lost demonstrate that Dpn functions cell-autonomously to maintain the self-renewal of larval NBs. Interestingly, in *dpn* mutant larvae, the premature loss of type I NBs mainly occurred within 48 hours ALH (with the exception of the MB NB). Zacharioudaki et al. [20] reported recently that Dpn and E(spl) proteins function redundantly to maintain NB self-renewal but have different temporal expression patterns. Dpn expression in NBs is activated at the newly hatched larval stage, whereas E(spl)mγ expression becomes obvious only when NBs start to divide at the 2^nd^ instar larval stage [20]. The difference in temporal expression patterns between Dpn and E(spl) proteins probably explains why loss of type I NBs occurred mainly within 48 hours ALH in *dpn* mutants. Interestingly, despite the redundant function of Dpn and E(spl) proteins in maintaining NB self-renewal, loss of Dpn alone resulted in the premature loss of MB NBs at late larval/early pupal stages, indicating that E(spl) proteins may not be involved in maintaining MB NBs at late larval/early pupal stages. Our findings as well as those of Zachariousdaki et al. [20] suggest that Dpn is required for maintaining NB self-renewal rather than NB formation or specification as was proposed by San-Juan and Baonza [21]. It is likely that differences in identifying and quantifying NBs at different developmental stages accounts for this discrepancy.

The role of Dpn in NB self-renewal is also supported by the observation that ectopic/over-expression of Dpn promoted non-dividing immature INPs and terminally dividing GMCs to enter self-renewing divisions, and prolonged the self-renewal of both types of NBs. These ectopic self-renewing GMCs and immature INPs, which normally do not express Dpn, may de-differentiate to acquire a NB-like fate and contribute to the increased number of NBs, similar to what has been observed in *brat*, *numb*and *klumpfuss* mutant type II NB lineages [4], [31]. However, type II NB lineages show more severe over-proliferation phenotypes than type I NB lineages in response to ectopic/over-expression of Dpn. The difference in degree of over-proliferation between the type I and type II NB lineages is likely related to intrinsic differences between the type I and type II NB daughters, rather than a difference in how Dpn itself is acting. Type I NBs produce GMCs that express genes such as *pros* and *ase* that limit proliferation [12], counteracting the pro-self-renewal function of Dpn. In contrast, type II NBs and immature INPs express the ETS family protein Pointed [16] but do not express Ase or Pros, making them particularly susceptible to the ectopic expression of genes, such as *dpn*, that promote self-renewal. We propose that the significantly enhanced proliferation of type II NB progeny in response to ectopic/increased Dpn expression is most likely due to a disparity in the inherent self-renewal potential of the type I and type II NB daughters.

The function of Dpn in maintaining NB self-renewal is consistent with mammalian Hes family proteins’ function in maintaining NSCs. In the developing mammalian nervous system, the loss of Hes1, Hes3, and Hes5 leads to accelerated neurogenesis and premature depletion of neuroepithelial cells and radial glial cells, whereas forced expression of Hes proteins maintains NSCs [32], [33], [34], [35], [36], [37].

### Dual Roles of Dpn in Type II NBs

While Dpn is expressed in both type I and type II NBs, our data showed that the loss of Dpn not only resulted in the premature loss of type II NBs at early larval stages, but also led to the ectopic expression of type I NB markers Ase and Pros in type II NBs when they exited quiescence, making type II NBs appear as type I-like NBs. This indicates Dpn has two roles in type II NBs: Dpn maintains NB self-renewal just as it does in type I NBs, and Dpn is also required to maintain type II NB identity. Moreover, it appears that Dpn’s role in maintaining type II NB identity is temporally restricted. Results from our study as well as others [21] showed that *dpn* mutant embryonic brains contained a comparable number of Dpn^+^ Ase^-^ NBs as wild type embryonic brains. In *dpn* mutant clones, our results showed that type II NBs did not ectopically express Ase even 4 days after clone induction when Dpn is no longer detectable. Therefore, it seems that Dpn’s function to suppress Ase expression is limited to a narrow temporal window during the reactivation of type II NBs at the 1^st^ instar larval stage. How might Dpn act to maintain type II NB identity? In mammals, Hes family proteins are well known for their roles in antagonizing the expression and/or activity of proneural genes (Ase is a member of the *achaete*-*scute* family of proneural genes) [38]. Negative interactions between *dpn* and the *achaete-scute complex (AS-C)* genes occur during *Drosophila* sex determination as well as neurogenesis [15], [39]. One potential model could be that a proneural protein(s) might be expressed in quiescent type II NBs and that Dpn is required to antagonize its expression and/or activity in order to promote type II NB fate when NBs exit quiescence. Since Dpn is expressed in both type I and type II NBs, we postulate that its role in maintaining type II NB fate is associated with the differential expression and/or activity of another, currently unidentified gene.

### Pros Likely Mediates the Premature Terminal Division of *dpn* Mutant NBs

Our work suggests that the premature loss of *dpn* mutant type I NBs could be mediated by Pros. This is supported by our findings that nuclear Pros precociously accumulates in *dpn* mutant type I NBs and that *dpn* mutant type I NBs are maintained even in adult brains in the absence of Pros. It has been shown that over-expressing Pros in embryonic and larval NBs is sufficient to induce ectopic nuclear Pros localization and terminal division [12], [40]. Therefore, one possibility is that Dpn negatively regulates *pros* expression. In the absence of Dpn, Pros expression increases, leading to the nuclear accumulation of Pros and thus premature terminal division. In type I NBs, dynamic cortical and cytoplasmic localization of Pros makes it difficult to compare the levels of Pros in wild type and *dpn* mutant type I NBs by immunostaining. However, ectopic Pros expression in *dpn* mutant type II NBs, which normally do not have Pros, provide evidence that Dpn negatively regulates Pros expression, either directly or indirectly. The existence of putative Dpn binding sites in the *pros* promoter suggests that Dpn could directly regulate *pros* expression [41]. Alternatively, Dpn could indirectly regulate *pros* by inhibiting the expression and/or activity of proteins, such as Ase, that promote *pros* expression. In support of this notion, it has been shown that mammalian Hes proteins can inhibit the expression of proneural proteins such as Mash1 in the developing cortex [42], whereas forced expression of the proneural protein Mash1 in neuroepithelial cells is sufficient to promote the expression of *Prox1*, the mammalian homolog of Pros that plays an anti-proliferative and pro-differentiation role in the developing mammalian hippocampus and retina [43], [44], [45], [46].

### Dpn does not Function Downstream of Notch in Larval NBs

Unlike the majority of mammalian Hes proteins or other members of the fly Hes family, which typically act downstream of Notch [34], [38], [47], results from this study as well as Zacharioudaki et al. [20] do not support a model in which Dpn functions as a direct target of Notch signaling in larval NBs as was proposed by San Juan and Baonza [21]. First, although our studies, as well as the work from other investigators [48], showed that the knockdown of Notch or disruption of Notch signaling led to premature loss of type II NBs and ectopic expression of Ase in type II NBs as was observed in *dpn* mutant larval brains, knockdown of Notch did not affect the expression of Dpn in type II NBs, which is consistent with previous findings [20]. Second, our data and those of Zacharioudaki et al. [20] showed that removing Dpn did not abolish the over-proliferation of type II or type I NBs caused by over-expression of activated Notch. Nor did reducing Notch expression exacerbate the loss of type II NBs in *dpn^7^* heterozygous animals. These genetic interaction data suggest that Dpn does not function downstream of Notch signaling. Thus, Dpn may be similar to the mammalian *Hes2* and *Hes3*, which are not transcriptionally regulated by Notch [49].Notch and Dpn likely employ distinct mechanisms to maintain the self-renewal and suppress Ase expression in type II NBs. Zacharioudaki et al. [20] showed that some E(Spl) proteins, particularly E(spl)mγ and m8, depend on Notch signaling for their expression in larval NBs. However, loss of E(Spl) proteins does not result in ectopic expression of Ase in type II NBs. Therefore, Notch must function through molecules, which are yet to be identified, to regulate Ase expression in type II NBs.

## Supporting Information

Figure S1 **Dpn protein perdures in** ***dpn^1^*** **mutant clones.** (A-B’) Dorsal views of a wild type (A) and *dpn^1^* mutant (B) 3^rd^ instar larval brain. Dpn is detected in wild type brains (A’) but not in *dpn^1^* mutant brains (B’). Note Ase-negative type II NBs (arrows in A) are absent in the *dpn^1^* mutant brain (B). (C-F’) *dpn^1^* mutant type I (C-D’) and type II (E-F’) NBs clones at 2 days (C, C’, E, E’) or 4 days (D, D’, F, F’) after clone induction. Dpn protein is detected at reduced levels in both type I (C, C’) and type II (E, E’) NBs at 2 days after clone induction, but not at 4 days after clone induction (D, D’, F, F’). Both type I (D, D’)and type II (F, F’) NBs remain present in *dpn^1^* mutant clones at 4 days after clone induction and type II NBs remain Ase^-^ (F, F’).
(PDF)

Figure S2 **Loss of type II NBs in** ***dpn*** **mutant 3^rd^ instar larval brains.** (A–A’) mCD8-GFP driven by *insc*-*Gal4* in combination with *ase*-*Gal80* labels the type II NB lineages in a 3^rd^ instar larval brain. Arrows indicate the Ase-negative type II NBs. (B–B’) In *dpn* mutant 3^rd^ instar larval brains, no NBs are labeled by mCD8-GFP driven by *insc*-*Gal4* with *ase*-*Gal80*.
(PDF)

Figure S3 ***dpn*** **mutant type II NBs ectopically express nuclear Pros at the 1^st^ instar larval stage.** (A–A”) Wild type type I NBs (arrows) express nuclear Pros at 9–12 hours ALH. (B–B”) Pros is not expressed in wild type type II NBs (arrowheads) at the same stage. (C–C”) Ectopic nuclear Pros in a remaining *dpn* mutant type II NB (arrowhead) at 9–12 hours ALH. Type II NBs were labeled with mCD8-GFP expression (in green) driven by *insc-Gal4 ase-Gal80*. NBs are stained with either Dpn (A–A”) or Mira (B–C”).
(PDF)

Figure S4 **Loss of Dpn does not affect the generation and specification of type II NBs in embryonic brains.** A wild type (A–A”) or a *dpn* mutant (B–B”) brain lobe at embryonic stage 14/15 (10.5–13.5 hrs AEL) is stained with Ase in red and Dpn in green. Arrows point to Dpn^+^Ase^-^ NBs. Wild type and *dpn* mutant brains contain similar numbers of Dpn^+^Ase^-^ NBs.
(PDF)

Figure S5 ***dpn*** **mutant MB NBs produce truncated lineages.** (A–B) Diagrams showing that individual MB NBs sequentially generate three distinct types (γ,α’/β’, and α/β) of MB neurons that target their axons to their corresponding lobes (B). (C–C’’) An adult wild type MARCM MB clone (in green) generated at the newly hatched larval stage has all three different types of MB neurons. The brain was counterstained with Fas II in red to label the γ and α/β axon lobes. (D–F) Adult *dpn^7^* mutant MB clones show various degrees of loss of late-born neurons. Among total 25 *dpn^7^* mutant clones examined, 17 clones show the loss of late-born α/β neurons as indicated by axon reduction in the center of the α/β lobe shown in a single focal plane (D–D’), 3 clones show a complete loss of α/β neurons (E), and 5 clones have only γ neurons (F). Insets in (C) and (F) show the cell body regions (outlined by dashed circles) of the corresponding clones. Note that the MB clones with only γ neurons contain much fewer MB neurons compared to wild type clones.
(PDF)

Figure S6 ***pros*** **mutant NBs persist in dpn mutant adult brains.** (A–B) *pros^v17^* (A) and *dpn pros^v17^* double mutant (B) type I NB clones in adult brains. Clones were labeled with mCD8 in red and stained for Dpn in green and Ase in blue. Numerous type I NBs are present in individual clones.
(PDF)

Figure S7 **Dpn overexpression in MARCM clones results in overproliferation in both type I and type II NB lineages.** (A) A wild type type II NB clone has a single Ase-negative NB (arrow) and a couple of Ase-negative immature INPs (arrowheads). (B) A type II NB clone overexpressing Dpn contains multiple NBs (arrows) and numerous immature INPs (arrowheads). (C) A wild type type I NB clone contains a single Ase-positive NB and a few Ase-positive GMCs (arrowheads). (D–D’) A type I NB clone overexpressing Dpn has a single NB (arrow) but an increased number of GMCs (arrowheads). (E–E’) A type I NB clone overexpressing Dpn contains multiple NBs (arrows) and increased number of GMCs.
(PDF)

Figure S8 **Ectopic Dpn expression causes immature INPs to become mitotically active.** (A–A’) phospho-histone H3 (p-H3) is not detected in Ase-negative immature INPs (arrows) in wild type type II NB lineages. (B–B’) Anti-p-H3 labels many immature INPs (arrows) when Dpn is ectopically expressed. Type II NB lineages are labeled by mCD8-GFP driven by *insc-Gal4* combined with *ase-Gal80*. NBs are outlined by dashed circles.
(PDF)

Figure S9 **Type II NBs are prematurely lost at the late 3^rd^ instar larval stage.** (A–A”) Type II NB lineages in control brains are labeled by mCD8-GFP driven by *GAL4^14–94^. GAL4^14–94^* also drives the expression of FLPase, which excises the stop codon and results in the expression of β-gal under the control of the constitutively active *actin* promoter. Through this approach, β-gal is expressed in type II NBs (arrows), which lack Ase, as well as type II NB progeny, which are also labeled by mCD8-GFP. (B–B”) In brains expressing Notch-RNAi, there is very little mCD8-GFP expression and there are no β-gal^+^ NBs, indicating Notch is necessary for maintaining NBs.
(PDF)
