# Supplementary figures and images for: The bHLH Repressor Deadpan Regulates the Self-renewal and Specification of Drosophila Larval Neural Stem Cells Independently of Notch
Source: PLoS One. 2012 Oct 8;7(10):e46724. doi: 10.1371/journal.pone.0046724 (PMC3466283; doi:10.1371/journal.pone.0046724)

Figure S1\_Zhu

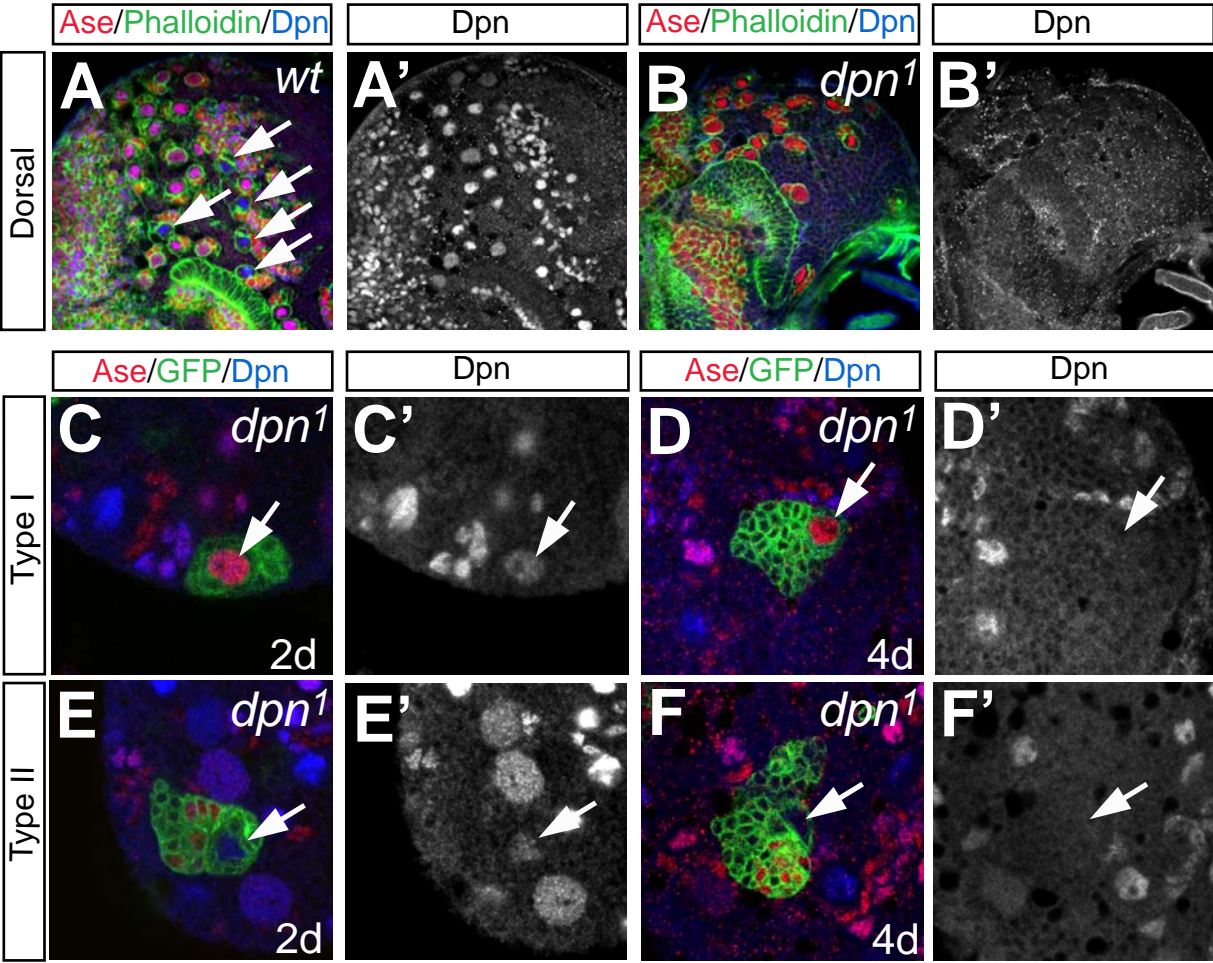

Supplement: Figure S1 — Dpn protein perdures in dpn1 mutant clones. (A-B’) Dorsal views of a wild type (A) and dpn1 mutant (B) 3rd instar larval brain. Dpn is detected in wild type brains (A’) but not in dpn1 mutant brains (B’). Note Ase-negative type II NBs (arrows in A) are absent in the dpn1 mutant brain (B). (C-F’) dpn1 mutant type I (C-D’) and type II (E-F’) NBs clones at 2 days (C, C’, E, E’) or 4 days (D, D’, F, F’) after clone induction. Dpn protein is detected at reduced levels in both type I (C, C’) and type II (E, E’) NBs at 2 days after clone induction, but not at 4 days after clone induction (D, D’, F, F’). Both type I (D, D’)and type II (F, F’) NBs remain present in dpn1 mutant clones at 4 days after clone induction and type II NBs remain Ase- (F, F’). (PDF) [file pone.0046724.s001.pdf]

Figure S2\_Zhu

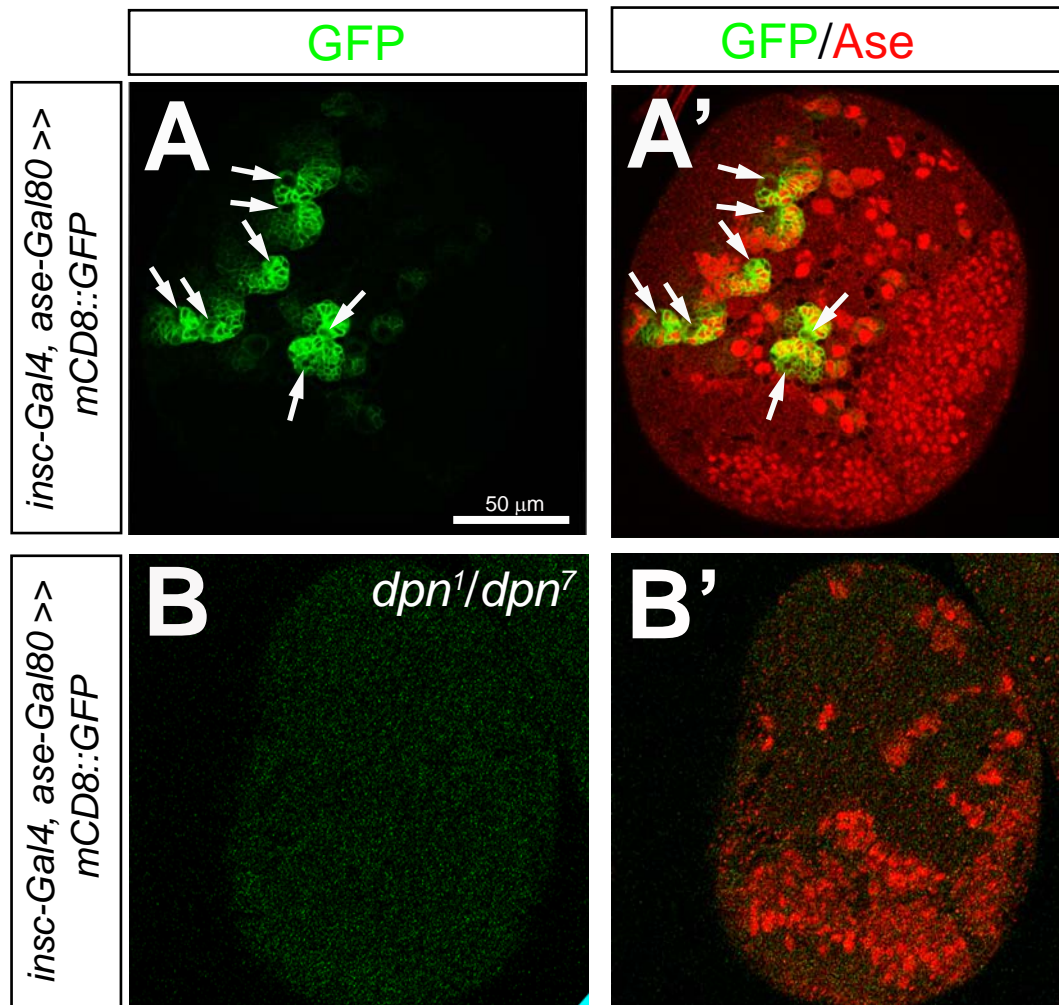

Supplement: Figure S2 — Loss of type II NBs in dpn mutant 3rd instar larval brains. (A–A’) mCD8-GFP driven by insc-Gal4 in combination with ase-Gal80 labels the type II NB lineages in a 3rd instar larval brain. Arrows indicate the Ase-negative type II NBs. (B–B’) In dpn mutant 3rd instar larval brains, no NBs are labeled by mCD8-GFP driven by insc-Gal4 with ase-Gal80. (PDF) [file pone.0046724.s002.pdf]

Figure S3\_Zhu

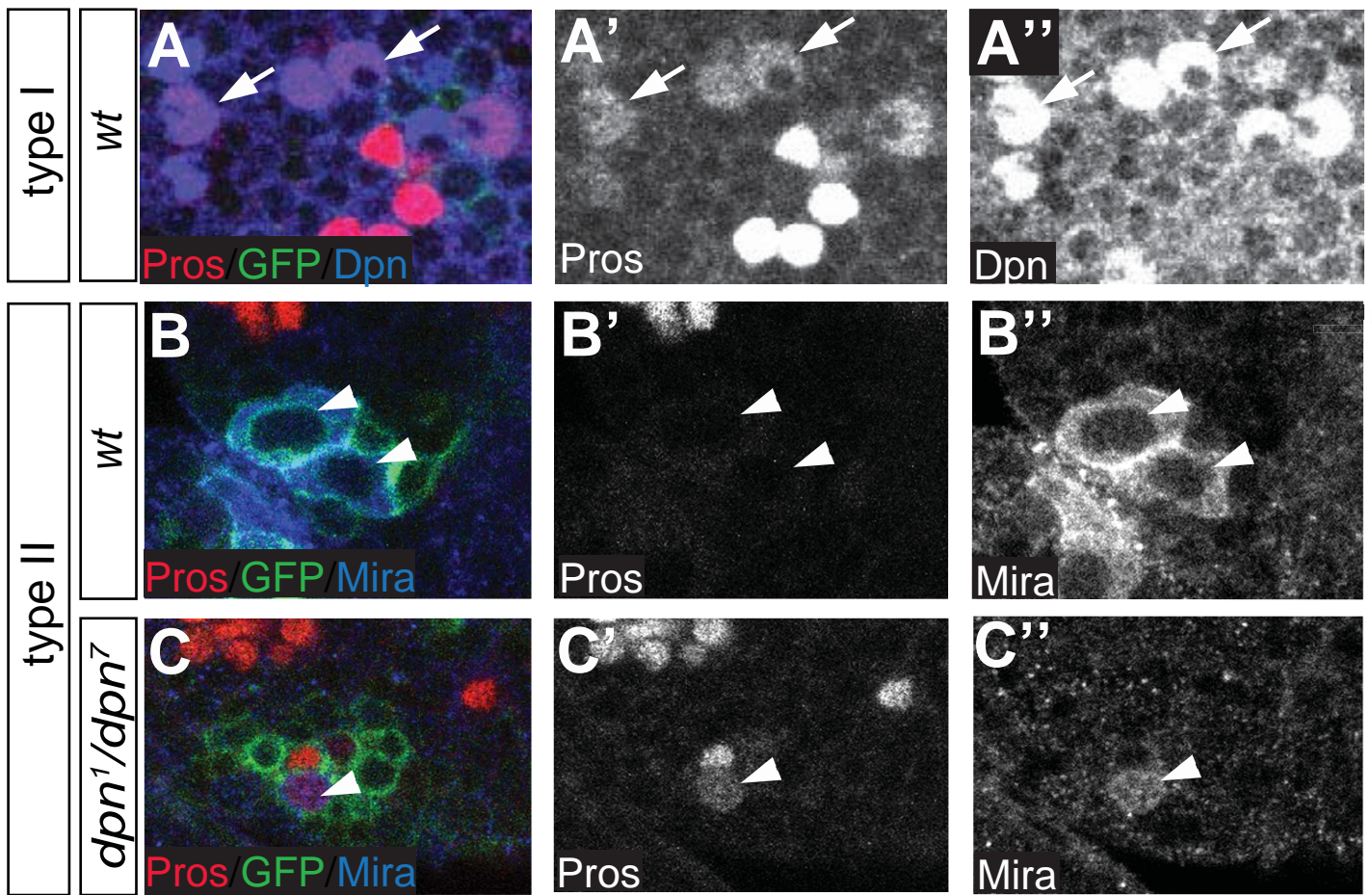

Supplement: Figure S3 — dpn mutant type II NBs ectopically express nuclear Pros at the 1st instar larval stage. (A–A”) Wild type type I NBs (arrows) express nuclear Pros at 9–12 hours ALH. (B–B”) Pros is not expressed in wild type type II NBs (arrowheads) at the same stage. (C–C”) Ectopic nuclear Pros in a remaining dpn mutant type II NB (arrowhead) at 9–12 hours ALH. Type II NBs were labeled with mCD8-GFP expression (in green) driven by insc-Gal4 ase-Gal80. NBs are stained with either Dpn (A–A”) or Mira (B–C”). (PDF) [file pone.0046724.s003.pdf]

Figure S4\_Zhu

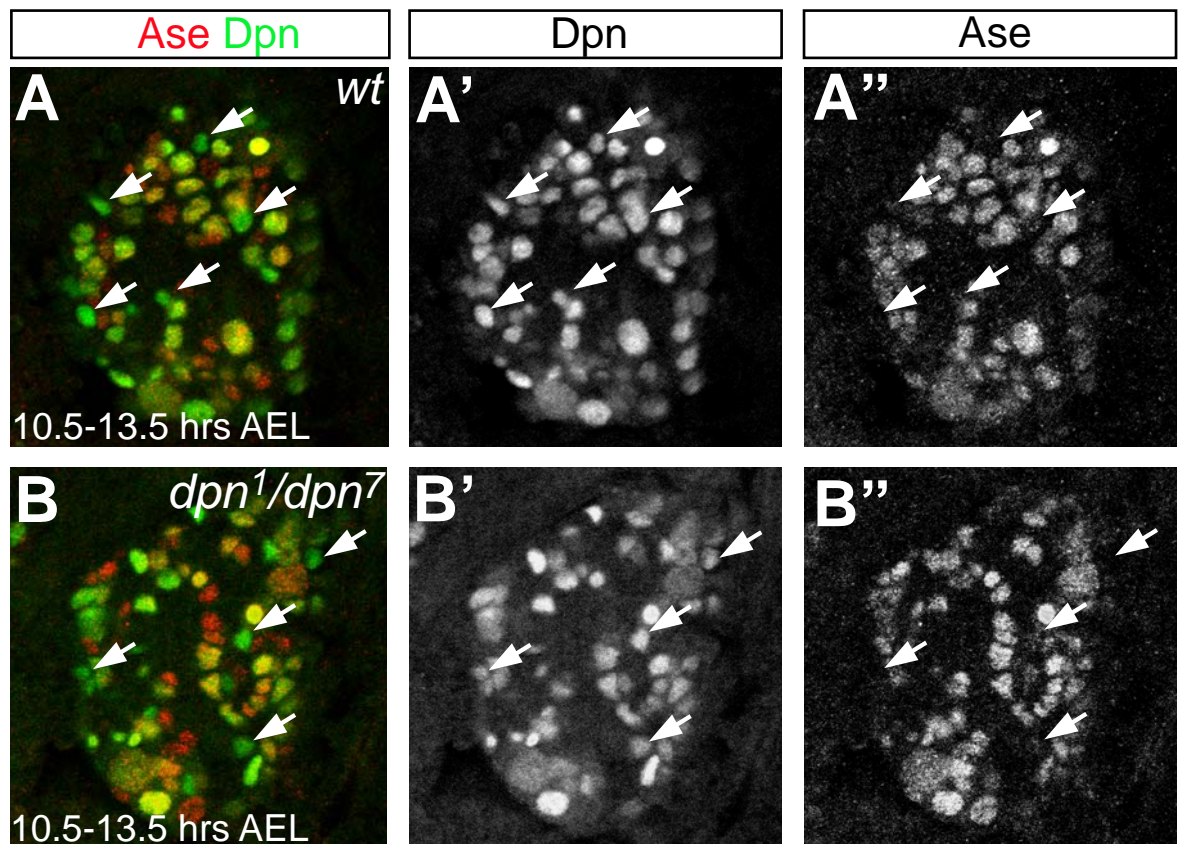

Supplement: Figure S4 — Loss of Dpn does not affect the generation and specification of type II NBs in embryonic brains. A wild type (A–A”) or a dpn mutant (B–B”) brain lobe at embryonic stage 14/15 (10.5–13.5 hrs AEL) is stained with Ase in red and Dpn in green. Arrows point to Dpn+Ase- NBs. Wild type and dpn mutant brains contain similar numbers of Dpn+Ase- NBs. (PDF) [file pone.0046724.s004.pdf]

Figure S5\_Zhu

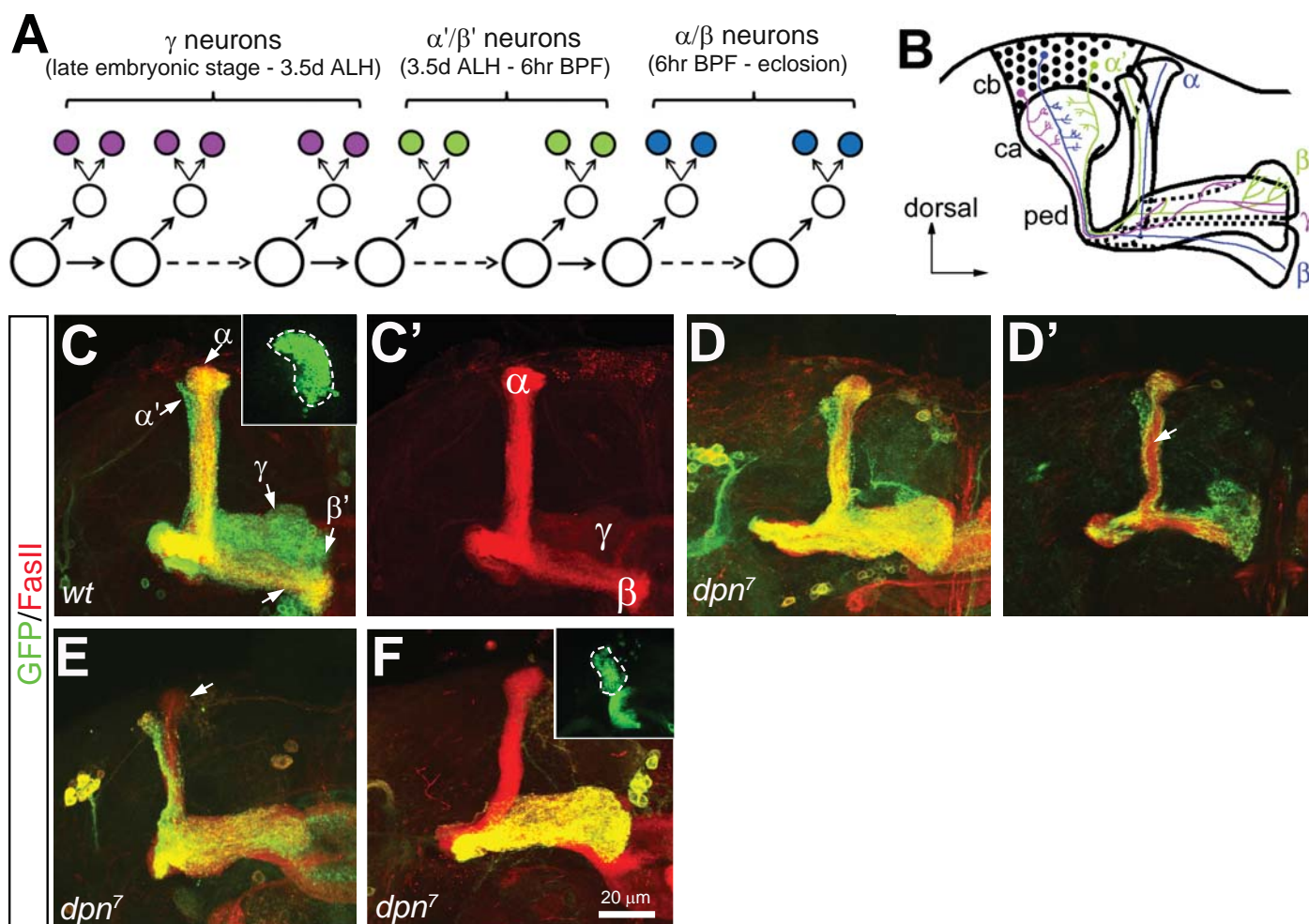

Supplement: Figure S5 — dpn mutant MB NBs produce truncated lineages. (A–B) Diagrams showing that individual MB NBs sequentially generate three distinct types (γ,α’/β’, and α/β) of MB neurons that target their axons to their corresponding lobes (B). (C–C’’) An adult wild type MARCM MB clone (in green) generated at the newly hatched larval stage has all three different types of MB neurons. The brain was counterstained with Fas II in red to label the γ and α/β axon lobes. (D–F) Adult dpn7 mutant MB clones show various degrees of loss of late-born neurons. Among total 25 dpn7 mutant clones examined, 17 clones show the loss of late-born α/β neurons as indicated by axon reduction in the center of the α/β lobe shown in a single focal plane (D–D’), 3 clones show a complete loss of α/β neurons (E), and 5 clones have only γ neurons (F). Insets in (C) and (F) show the cell body regions (outlined by dashed circles) of the corresponding clones. Note that the MB clones with only γ neurons contain much fewer MB neurons compared to wild type clones. (PDF) [file pone.0046724.s005.pdf]

Figure S6\_Zhu

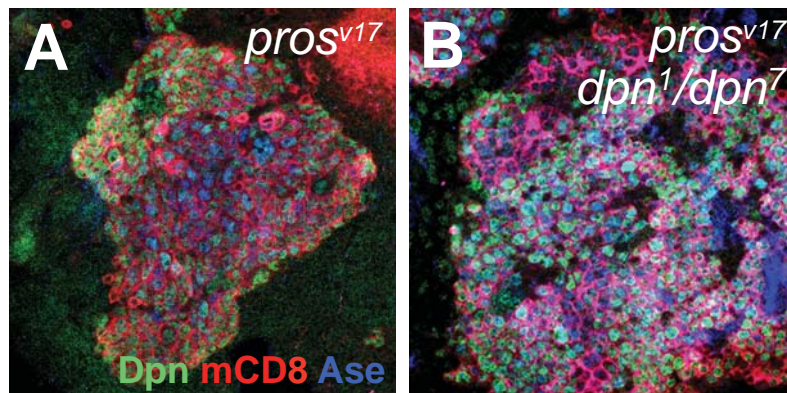

Supplement: Figure S6 — pros mutant NBs persist in dpn mutant adult brains. (A–B) prosv17 (A) and dpn prosv17 double mutant (B) type I NB clones in adult brains. Clones were labeled with mCD8 in red and stained for Dpn in green and Ase in blue. Numerous type I NBs are present in individual clones. (PDF) [file pone.0046724.s006.pdf]

Figure S7\_Zhu

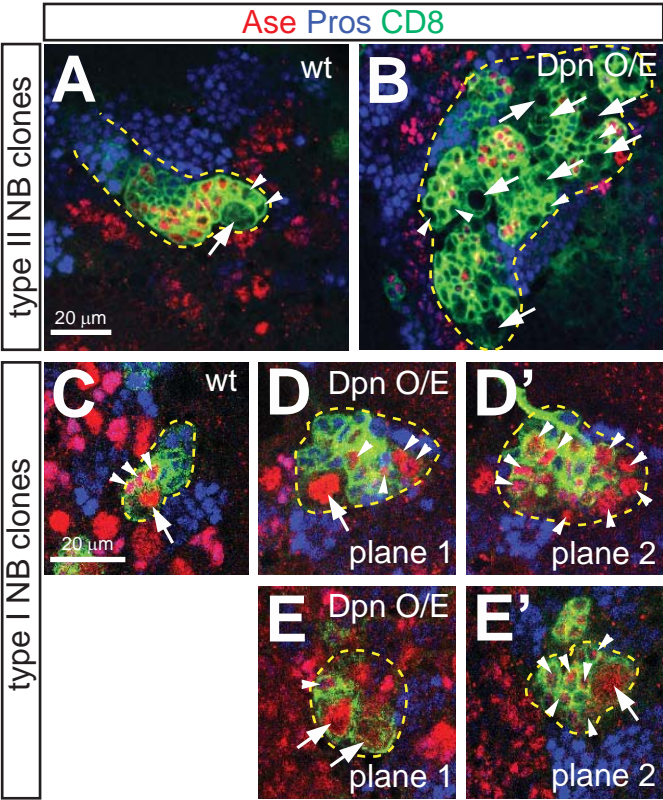

Supplement: Figure S7 — Dpn overexpression in MARCM clones results in overproliferation in both type I and type II NB lineages. (A) A wild type type II NB clone has a single Ase-negative NB (arrow) and a couple of Ase-negative immature INPs (arrowheads). (B) A type II NB clone overexpressing Dpn contains multiple NBs (arrows) and numerous immature INPs (arrowheads). (C) A wild type type I NB clone contains a single Ase-positive NB and a few Ase-positive GMCs (arrowheads). (D–D’) A type I NB clone overexpressing Dpn has a single NB (arrow) but an increased number of GMCs (arrowheads). (E–E’) A type I NB clone overexpressing Dpn contains multiple NBs (arrows) and increased number of GMCs. (PDF) [file pone.0046724.s007.pdf]

Figure S8\_Zhu

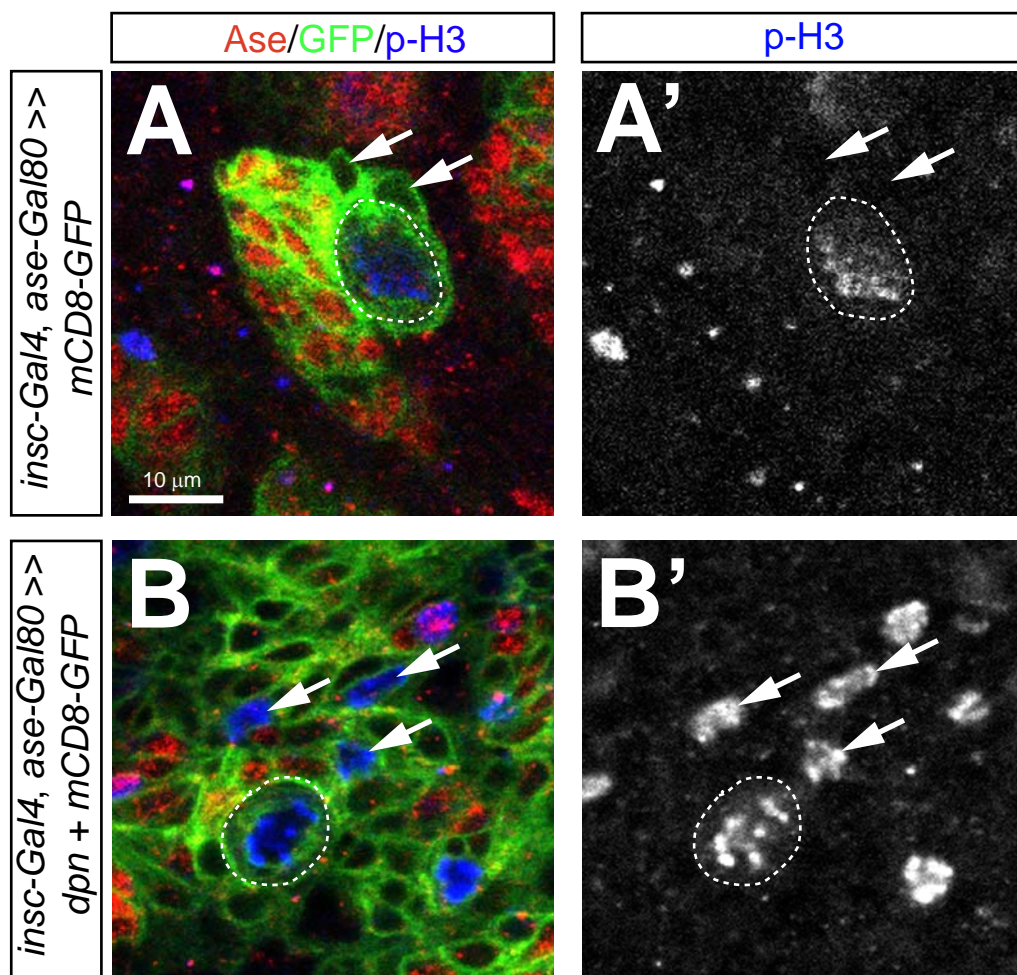

Supplement: Figure S8 — Ectopic Dpn expression causes immature INPs to become mitotically active. (A–A’) phospho-histone H3 (p-H3) is not detected in Ase-negative immature INPs (arrows) in wild type type II NB lineages. (B–B’) Anti-p-H3 labels many immature INPs (arrows) when Dpn is ectopically expressed. Type II NB lineages are labeled by mCD8-GFP driven by insc-Gal4 combined with ase-Gal80. NBs are outlined by dashed circles. (PDF) [file pone.0046724.s008.pdf]

Figure S9\_Zhu

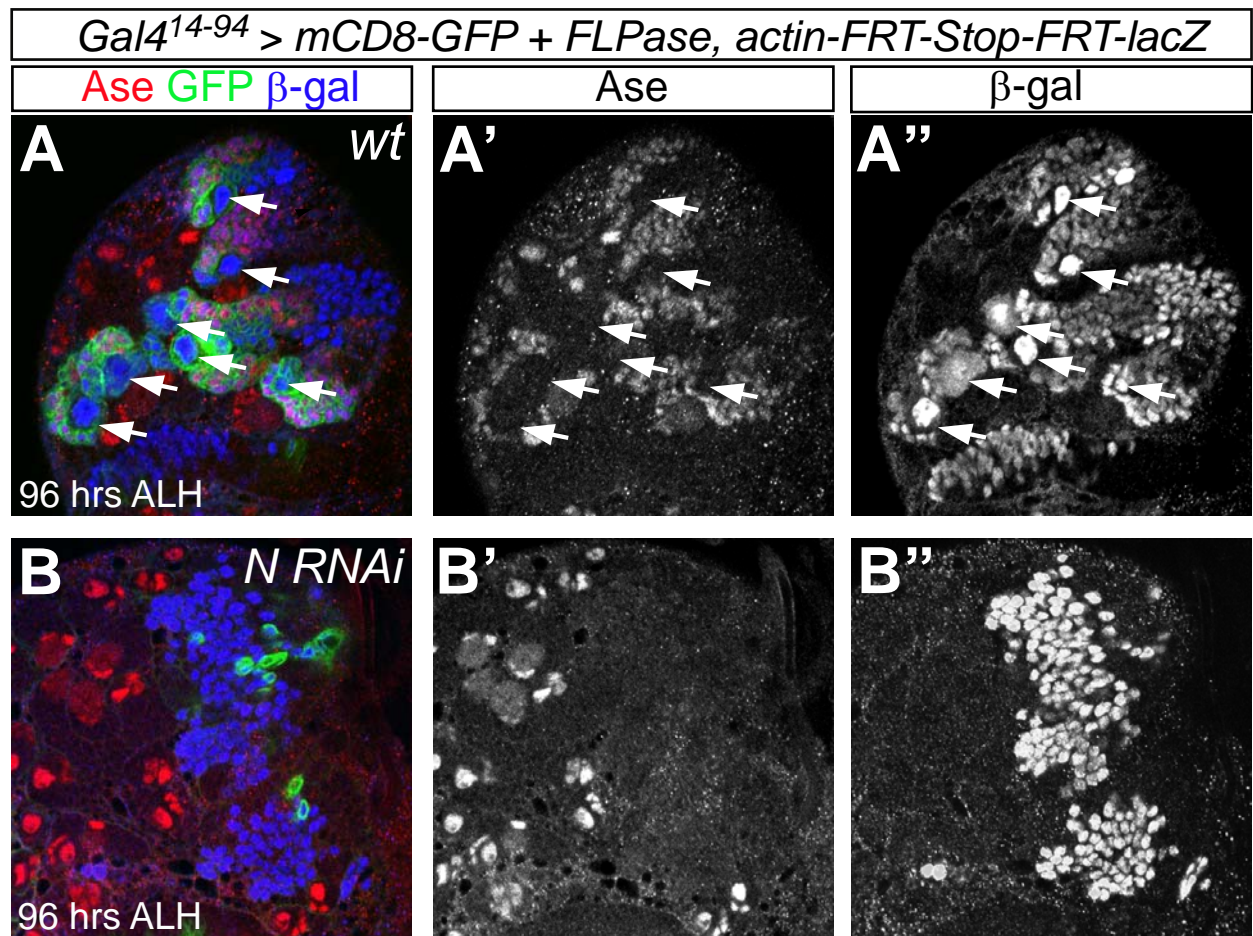

Supplement: Figure S9 — Type II NBs are prematurely lost at the late 3rd instar larval stage. (A–A”) Type II NB lineages in control brains are labeled by mCD8-GFP driven by GAL414–94. GAL414–94 also drives the expression of FLPase, which excises the stop codon and results in the expression of β-gal under the control of the constitutively active actin promoter. Through this approach, β-gal is expressed in type II NBs (arrows), which lack Ase, as well as type II NB progeny, which are also labeled by mCD8-GFP. (B–B”) In brains expressing Notch-RNAi, there is very little mCD8-GFP expression and there are no β-gal+ NBs, indicating Notch is necessary for maintaining NBs. (PDF) [file pone.0046724.s009.pdf]
